# Engineered whole cut meat-like tissue by the assembly of cell fibers using tendon-gel integrated bioprinting

**DOI:** 10.1038/s41467-021-25236-9

**Published:** 2021-08-24

**Authors:** Dong-Hee Kang, Fiona Louis, Hao Liu, Hiroshi Shimoda, Yasutaka Nishiyama, Hajime Nozawa, Makoto Kakitani, Daisuke Takagi, Daijiro Kasa, Eiji Nagamori, Shinji Irie, Shiro Kitano, Michiya Matsusaki

**Affiliations:** 1 Division of Applied Chemistry, Graduate School of Engineering, Osaka University, Osaka, Japan; 2 Joint Research Laboratory (TOPPAN INC.) for Advanced Cell Regulatory Chemistry, Graduate School of Engineering, Osaka University, Osaka, Japan; 3 Department of Anatomical Science, Hirosaki University Graduate School of Medicine, Hirosaki, Japan; 4 NH Foods, Ltd., Tsukuba, Japan; 5 Kirin Central Research Institute, Kirin Holdings Company, Ltd., Fujisawa, Japan; 6 Biomedical Business Center, Healthcare Business Group, Ricoh Company, Ltd., Kawasaki-shi, Japan; 7 Solution Planning, Product Solution Technologies, Production Printing, Industrial Solutions, Ricoh Japan Corporation, Tokyo, Japan; 8 Department of Biomedical Engineering, Faculty of Engineering, Osaka Institute of Technology, Osaka, Japan; 9 TOPPAN INC., Technical Research Institute, Saitama, Japan

**Keywords:** Biomaterials - cells, Stem-cell biotechnology, Tissue engineering, Biomedical engineering, Biomaterials - cells

## Abstract

With the current interest in cultured meat, mammalian cell-based meat has mostly been unstructured. There is thus still a high demand for artificial steak-like meat. We demonstrate in vitro construction of engineered steak-like tissue assembled of three types of bovine cell fibers (muscle, fat, and vessel). Because actual meat is an aligned assembly of the fibers connected to the tendon for the actions of contraction and relaxation, tendon-gel integrated bioprinting was developed to construct tendon-like gels. In this study, a total of 72 fibers comprising 42 muscles, 28 adipose tissues, and 2 blood capillaries were constructed by tendon-gel integrated bioprinting and manually assembled to fabricate steak-like meat with a diameter of 5 mm and a length of 10 mm inspired by a meat cut. The developed tendon-gel integrated bioprinting here could be a promising technology for the fabrication of the desired types of steak-like cultured meats.

## Abstract (web summary)

Mammalian cell-based cultured meat has mostly been unstructured, leaving a demand for artificial steak-like meat. Here the authors present an assembled steak-like tissue of bovine skeletal muscle, adipose tissue, and blood capillary tissue fabricated by tendon-gel integrated printing technology.

## Introduction

Over the past decade, cultured meat has drawn tremendous attention from the standpoints of ethics, economics, the environment, and public health, although it is still under debate^1^. More recently, meat analogs that taste like meat but are based on plant proteins have been released commercially^1,2^. Although challenges remain unlike with meat analogs, cultured meat is highly sought after due to the possibility of imitating real meat through the manipulation of flavor, muscle/adipose cells’ ratio, and texture^3,4^.

Bovine cells for cultured meat can currently be secured by two approaches^5,6^. One is following the obtention of edible muscle tissues from cattle, their cells are separated into each type such as muscle satellite cells, adult stem cells, and multipotent stem cells, which are then cultured to increase the number of cells. The other is from transforming somatic cells into induced pluripotent stem cells (iPSCs) and differentiating them into each cell type. Primary cultured stem cells, particularly muscle satellite cells, maintain their differentiation capability within 10 passages and thus have a limited number of divisions^7^. But, they should still be safe and acceptable for consumption.

Since Post and coworkers unveiled bovine cell fiber-based hamburger, various types of cultured meat have been demonstrated. However, cultured steak with a composition and a structure similar to real steak, comprising mostly adipose cells and aligned muscle cells, is still challenging^4,8,9^. Various tissue engineering techniques could be applied, such as cell sheet engineering^10,11^, cell fiber engineering^12^, cell culture on a 3D-printed scaffold^13^, and 3D cell printing^14,15^ for mimicking the structural characteristics of steak. Among them, 3D cell printing is promising due to its advantages of scalability and controllability of structure and composition^16^. Especially, a supporting bath-assisted 3D printing (SBP) technique where ink is dispensed inside a gel or a suspension with thixotropy is noteworthy. Under shear forces, the viscosity of a gel or a suspension becomes of low viscosity, enabling the ink dispensing, and it returns to a high viscosity when the shear force is released, maintaining the printed form^17^. Since the SBP is able to overcome not only the restricted ink viscosity range but also the drying problem during prolonged printing in extrusion-based 3D printing in the air-interfaced environment, several studies over the past five years have shown the feasibility of complex tissue fabrication^18–24^.

**Fig. 1:**
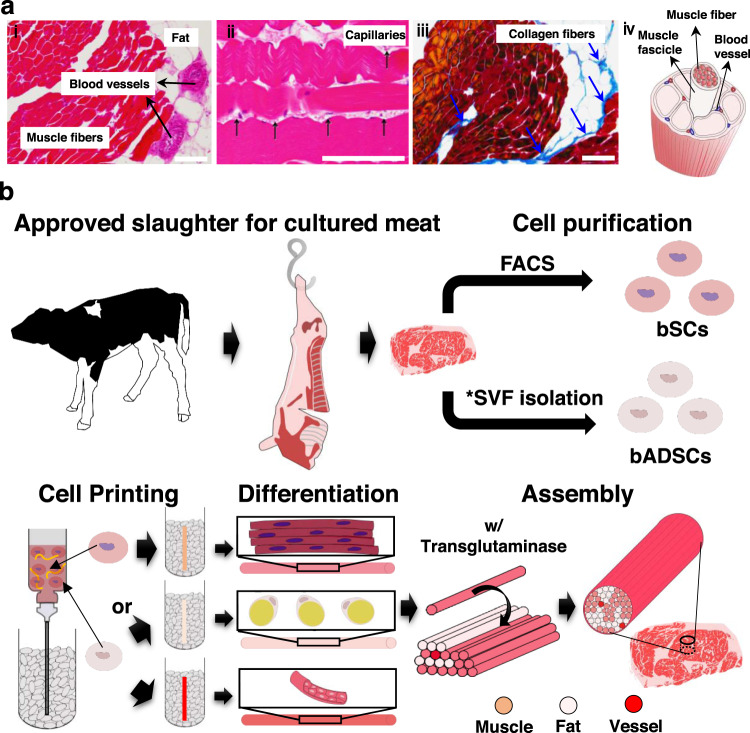
Overview of the work. **a** Structure of steak. (i, ii) H&E- and (iii) Azan-stained images of a piece of steak. Representative images from three independent experiments are shown. All scale bars denote 100 μm (iv) Schematic of a hierarchical structure in muscle. **b** Schematic of the construction process for cultured steak. The first step is cell purification of tissue from cattle to obtain bovine satellite cells (bSCs) and bovine adipose-derived stem cells (bADSCs). The second is supporting bath-assisted printing (SBP) of bSCs and bADSCs to fabricate the muscle, fat, and vascular tissue with a fibrous structure. The third is the assembly of cell fibers to mimic the commercial steak’s structure. *SVF stromal vascular fraction.

Steak meat has an aligned structure of skeletal muscle fascicles with a diameter from around 900 μm to 2.3 mm^25^, depending on age and animal parts, formed by assembled skeletal muscle fibers, connected to the tendon for its shrinkage and relaxation movements. The muscle fibers are covered with basement membrane and the muscle fascicles are surrounded by fat together with blood capillaries (Fig. 1a). The component ratio and location of the muscle, adipose tissues, and blood capillaries are significantly different according to the meat type and its country of origin. For example, red meat in the rump of Japanese Wagyu has only 10.7% adipose tissues, whereas the sirloin of the Wagyu has 47.5%^26^. Accordingly, the development of a methodology for assembling the three types of fibers with the desired location, ratio, and amount will be a key manufacturing technology of cultured steak.

Here, we demonstrate a three-step strategy for the construction of engineered steak-like meat: (1) collection of edible bovine satellite cells (bSCs) and bovine adipose-derived stem cells (bADSCs) from beef meats and their subsequent expansion, (2) development of the tendon-gel-integrated bioprinting (TIP) for the fabrication of cell fibers and their subsequent differentiation to skeletal muscle, adipose, and blood capillary fibers, and (3) assembly of the differentiated cell fibers to construct engineered steak-like meat by mimicking the histological structures of an actual beef steak (Fig. 1b). Since the tendon is a key tissue for the muscle fiber alignment and maturation, we fabricated tendon gels by TIP to enable their consecutive connection with the muscle cell fibers, inducing the formation of aligned matured muscle fibers. In this study, a total of 72 fibers comprising 42 muscle, 28 adipose, and 2 blood capillaries were constructed by TIP. They were subsequently assembled to fabricate steak-like meat with a diameter of 5 mm and a length of 10 mm inspired by the histological images of an actual Wagyu beef steak. TIP is expected to become a powerful approach for constructing engineered steak-like meat with desired location, component ratio, and amount of the three types of fibers.

## Results

### Verification of the differentiation conditions for the extracted bSCs and bADSCs

**Fig. 2:**
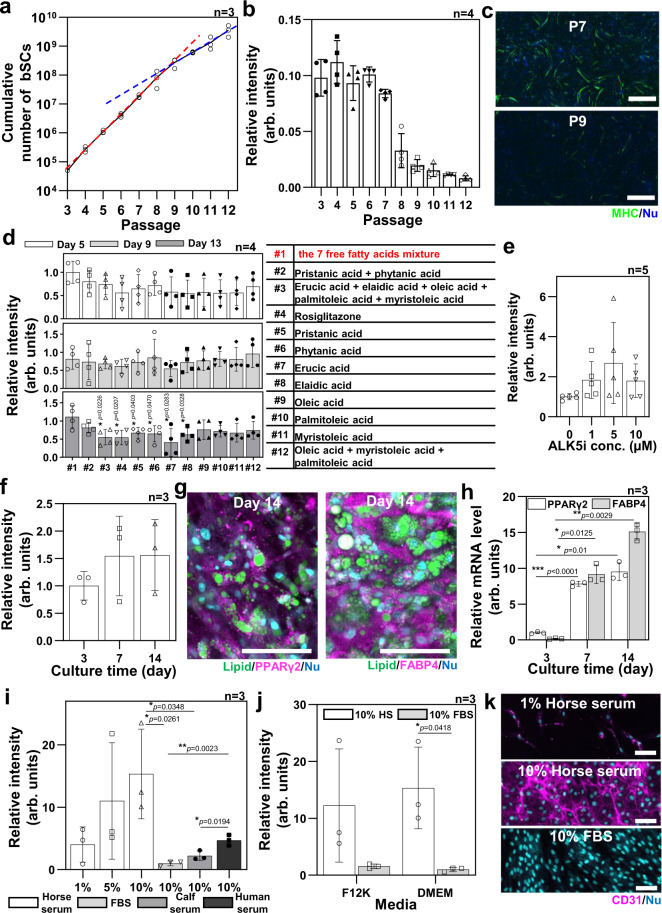
Verification of purified bovine stem cells. **a, b** Proliferation rate (*n* = 3 independent samples) (**a**) and differentiation ratio (*n* = 4 independent areas examined over three independent samples) on day 5 of differentiation (**b**) of bSCs from passage 3 (P3) to P12 cultured on a tissue culture plate. Red and blue lines are a slope from P3 to P8 and from P8 to P12, respectively. **c** Representative immunofluorescence images of differentiation induced bSCs at P7 and P9 stained for myosin II heavy chain (MHC) (green) and nucleus (blue) from at least three independent experiments. Scale bars, 1 mm. **d** Adipogenesis ratio (left) of 3D gel-drop-cultured bADSCs derived by 12 combinations of free fatty acids (middle) in DMEM on days 5, 9, and 13 (*n* = 4 independent experiments, two-way ANOVA paired for the time and unpaired for the treatment with a Tukey’s HSD post test). **e, f** Lipid-droplet production in 3D-cultured bADSCs, depending on the concentration of ALK5i on day 7 (**e**) and culture day (**f**) in the #1 combination of free fatty acids and 5 μM (*n* = 5 (e) and 3 (f) independent experiments, unpaired (e) and paired (f) one-way ANOVA with a Tukey’s HSD post test). **g, h** Representative immunofluorescence images from three independent experiments (**g**) and mRNA expression levels (**h**) of 3D bADSCs tissue cultured with the media containing seven free fatty acid mixture (#1) and 5 μM ALK5i (*n* = 3 independent experiments, paired one-way ANOVA with a Tukey’s HSD post test). **i, j** CD31 immunostaining quantitation of bADSCs in 2D, depending on serum conditions in DMEM (**i**) and base media (**j**) on day 7 (*n* = 3 independent experiments, unpaired one-way ANOVA with a Tukey’s HSD post test (i) and unpaired two-way ANOVA with a Šidák post test (j)). **k** Representative immunofluorescence images of bADSCs depending on serum conditions on day 7 stained for CD31 (magenta) and nucleus (blue) from three independent experiments. Scale bars, 100 μm. The used bADSCs were extracted from subcutaneous fat. *p<0.05, **p<0.01, ***p<0.001; error bars represent mean ± s.d. Source data are provided as a Source Data file.

The bSCs were extracted from the masseter muscle of 27-month-old Japanese black cows obtained from a slaughterhouse using a method modified from a previously reported one^7^. The crude cell fraction separated from the beef meat by collagenase treatment was cultured until passage 3 (P3) for cell sorting. The CD31^−^, CD45^−^, CD56^+^, and CD29^+^ cells were isolated by fluorescence-activated cell sorting (Supplementary Fig. 1), in which Pax7^+^ bSCs were around 80%. 2D culture of the isolated bSCs was performed to evaluate their proliferation and differentiation potentials into muscle cells even after prolonged passaging. After seeding, the bSC passage was incremented after each cell detachment by trypsinization every two days. The proliferation medium contained not only fetal bovine serum (FBS) and basic fibroblast growth factor but also a p38 inhibitor to maintain the differentiation potential of the proliferating bSCs^7^. The number of seeded bSCs doubled approximatively once per day until P8, and about once every two days thereafter (Fig. 2a). The differentiation was induced after two days of seeding by switching the basic medium for a differentiation media containing 2% horse serum (HS), which is a well-known differentiation-induction method for muscle cells. The cells were immunostained with the antibody of myosin II heavy chain (MHC) after five days of differentiation induction. We quantified the differentiation capacity regarding the passage number of the seeded bSCs by calculating the ratio of DAPI fluorescence intensity between MHC^+^ and MHC^-^ cells from fluorescence images (Supplementary Fig. 2). The bSCs from P3 to P7 expressed a comparable differentiation level, but the differentiation capability of bSCs above P8 significantly decreased (Figs. 2b and 2c). Therefore, we conducted experiments using cells prior to P8.

Next, 3D culture with collagen microfibers (CMF)/fibrin gel was performed for assessing the adipogenic differentiation potential of the bADSCs in a variety of media conditions since it is known that the adipogenesis of adipose-derived stem cells (ADSCs) in 3D culture is higher than in 2D culture^27^ and that the differentiation-factor efficiency relies on species^28^. Conventional human adipogenic factors like insulin, rosiglitazone, or troglitazone were thus first found with limited lipogenesis (Supplementary Fig. 3), leading to the direct addition of free fatty acids (pristanic acid, phytanic acid, erucic acid, elaidic acid, oleic acid, palmitoleic acid, and myristoleic acid) to the culture medium following previous published methods^29,30^. The different combinations of the seven aforementioned free fatty acids were also compared and the results showed a higher lipogenesis by lipid storage into the cytoplasmic vesicles of the bovine preadipocytes for the medium containing all the seven free fatty acids (Fig. 2d and Supplementary Fig. 4). To further increase the lipogenesis until reaching a matured bovine adipocyte state, the transforming growth factor (TGF) type I receptor activin-like kinase 5 inhibitor (ALK5i) effect was evaluated because this factor is an inhibitor of the TGF-β receptor ALK5 and TGF‐β family ligands, contained in the 10% FBS of the culture medium, which is known to inhibit both adipogenesis and adipocyte hypertrophy^31^. The TGF‐β family also includes myostatin, which is expressed by the myocytes to impair adipogenesis^32^. In the context of future coculture between bovine myoblasts and adipocytes, ALK5i appeared relevant for further inducing the adipogenic potential of the culture medium containing the seven free fatty acids. Several concentrations were thus assessed from 1 to 10 µM. The results showed a tendency to a higher lipogenesis with 5 µM ALK5i (Fig. 2e and Supplementary Fig. 5a). The lipogenesis of the bADSCs then increased progressively between 3 and 14 days of differentiation (Fig. 2f and Supplementary Fig. 5b).

In addition to lipogenesis, further investigations concerning the two adipogenic markers PPARγ2 and FABP4 were conducted to evaluate the adipogenesis. The immunostaining of PPARγ2, one of the most important transcription factors for fat cell differentiation, showed a slight expression inside the bADSCs after three days of differentiation, which was specifically found in the nuclei due to their role as an early transcription factor, inducing the other adipogenic maturation genes^33^ (Supplementary Fig. 6). This location in the nuclei is less observed after 14 days of differentiation, implying a more matured state of the bADSCs (Fig. 2g and Supplementary Fig. 6). Concerning FABP4, a late specific adipogenic marker necessary for trafficking fatty acids to the membrane for efflux^34^, its expression location was observed in the cytoplasm, increasing following the differentiation duration with a particularly high expression observed in the unilocular mature adipocytes (Fig. 2g and Supplementary Fig. 6). The increase of both early and late marker expressions was confirmed by the RNA (qPCR) analysis, which highlighted the significant linear-increasing expression profile of the PPARγ2 marker, followed by the FABP4 to a higher extent testifying the mature state of the bADSCs-derived adipocytes (Fig. 2h).

Recently, ADSCs have been considered to be a useful cell source for angiogenesis in tissue engineering, but unlike human ADSCs, there are no reports on endothelial differentiation of bADSCs^35,36^. Knowing that they can lose their differentiation potentials during ADSCs culture expansion^37^, bADSCs were thus used at P1 to evaluate their endothelial differentiation in different conditions. Horse serum (HS) was surprisingly found to be a significant inducer of the CD31 expression, an endothelial cell marker (4 and 15 times more for 1 and 10% HS, compared with the 10% FBS condition), independently of the medium used, DMEM or F12K (Fig. 2i–j and Supplementary Fig. 7). The human serum also provided an enhanced endothelial differentiation, compared with the FBS condition, but was impaired by the low cell proliferation observed (Supplementary Fig. 7). In addition to CD31 expression, the tubulogenesis was confirmed by culturing the seven-day differentiated cells on Matrigel in media containing 10% HS (Supplementary Fig. 8). The DMEM + 10% HS was then used for the endothelial differentiation from bADSCs in this study.

### Bovine muscle fiber fabrication by supporting bath-assisted 3D printing

To organize the isolated bSCs into a cell fiber, we utilized a supporting bath-assisted 3D printing (SBP) consisting of a bioink dispensed inside a supporting bath usually composed of a hydrogel slurry that ensures the printed-structure stability in the *z* axis. Several studies have also demonstrated its promise in cell printing for its high-shape fidelity even on complex or soft structures, and for its stable printing during prolonged operation^20,21,23,24^. We selected gelatin and gellan gum as supporting bath materials, due to their edible, removable, and cell-compatible properties. Gelatin is a gel at room temperature (RT) and a liquid at 37 °C, therefore, it is easy to remove after printing by incubation at 37 °C^19^. Gellan gum hydrogel is also known to dissolve in 50 mM Tris-HCl buffer at pH 7.4 and at 37 °C^38^. Hydrogel slurry was fabricated by homogenizing bulk hydrogel of gellan gum and gelatin, in which the average particle sizes are 44 μm and 70 μm, respectively, and their thixotropy behavior was confirmed (Supplementary Fig. 9).

**Fig. 3:**
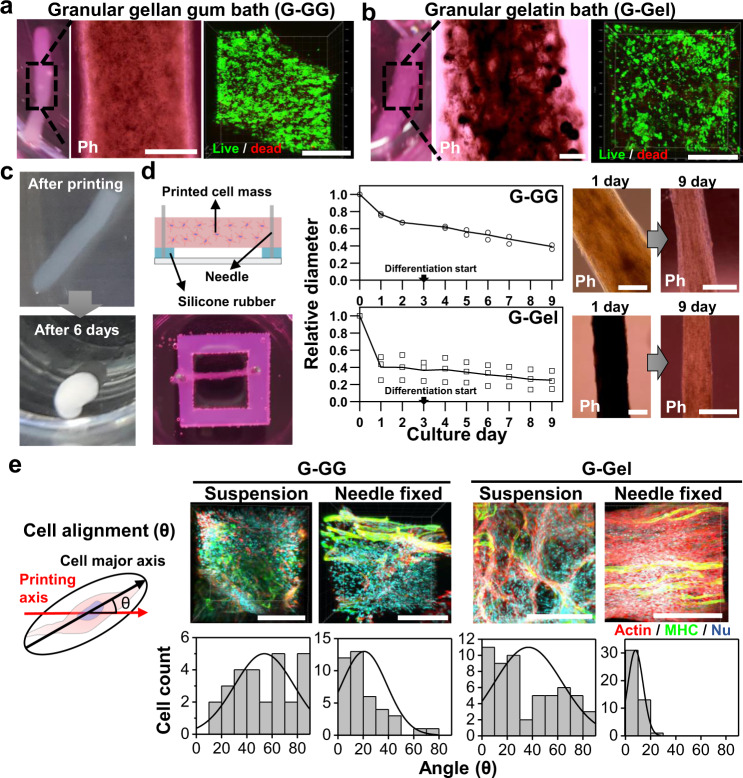
The characterization of bSC tissue fabricated by SBP. **a, b** Optical (left), phase contrast (middle), and fluorescence (right, green: live cells and red: dead cells) images of the bSC tissues printed inside granular gellan gum (G-GG) (**a**) and granular gelatin (G-Gel) (**b**) followed by bath removal. Scale bars, 500 µm. **c** Shape change of bSC tissue fabricated by SBP inside G-Gel from the fibrous form right after printing and bath removal to globular form on day 6 of suspension culture. **d** Schematic (left), size change in accordance with culture day (middle), and phase-contrast images (right) of needle fixed culture of printed bSCs tissues. Error bars represent mean ± s.d. Scale bars, 500 µm. **e** 3D-fluorescence images (upper, red: actin and green: MHC) and cell alignment measurements (lower) of the bSC tissues printed inside G-GG and G-Gel and in suspension and needle-fixed cultures on day 3 of differentiation (after six days), respectively. Representative images from at least two independent experiments are shown. Scale bars, 200 µm. Source data are provided as a Source Data file.

First, we tried to print the bioink containing bSCs, fibrinogen, and Matrigel solution in the culture medium into the supporting bath mixed with granular particles of gelatin (G-Gel) or gellan gum (G-GG) and thrombin for the fabrication of a fibrous muscle fiber mimicking the bundle of muscle fiber in steak (Supplementary Movies 1 and 2). With the confirmation of the gel formation followed by the removal of supporting baths, high cell viability was observed for nine days after printing in both the G-Gel and G-GG by live/dead staining (Figs. 3a, 3b and Supplementary Fig. 10).

When the printed cell fiber was cultured in suspension, the fiber collapsed to a globular form (Fig. 3c). Studies related to muscle tissue engineering have implied that an anchor structure enables the 3D muscle tissues to not only maintain their initial shape but also to improve the cell alignment, fusion, and differentiation against the muscle fiber’s contraction^15,39–43^. We placed a printed cell fiber onto a silicone rubber and anchored it with needles fastening both ends to withstand cell contractions (Fig. 3d, left). With the needle-fixed culture, the cell fibers printed inside G-GG and G-Gel retained the fibrous structure, but the diameter shrank by around 60% in G-GG and 80 % in G-Gel at day 9 of culture (Fig. 3d, right). It would be reasonable to suppose that the size decrease was caused by the alignment and fusion of bSCs along with the enzymatic decomposition of fibrin gel by the proteases secreted from the cells^44,45^. We also took immunofluorescence images inside the cell fibers to examine the cellular behavior w/ and w/o needle anchoring on day 3 of differentiation. To quantify the improvement in terms of muscle maturation, the cell alignment, i.e., the angle setting for the straight line between needles, was measured from the immunofluorescence images (Fig. 3e, left). The results showed that the cells in the cell fiber of the suspension culture were randomly oriented, regardless of the type of supporting baths, while in the needle-fixed culture the cells in the cell fiber printed inside G-Gel were anisotropically oriented compared with those of G-GG (Fig. 3e, right). We postulated that the difference in the degree of alignment between G-GG and G-Gel arises from the hindrance of cell behaviors by some residual substances that might exist inside or on the printed cell fibers. This substance was found to be the residual G-GG in the cell fiber (Supplementary Fig. 11), which may not be degraded or dissolved, limiting the cells in their ECM remodeling required to migrate and fuse with the other cells. On the other hand, G-Gel is easily dissolved at 37 °C and may be degraded by proteases, enabling an active cell behavior, despite the possible residues that might remain in the printed cell fiber. Printing bSCs inside G-Gel and anchoring them are the essential steps for the fabrication of the muscle cell fiber, but the anchoring method may not be appropriate for the scale-up. Therefore, we developed a modified SBP to include a part that can be simultaneously anchored by the printed cell fiber.

### Fabrication of muscle, fat and vascular cell fibers by TIP

**Fig. 4:**
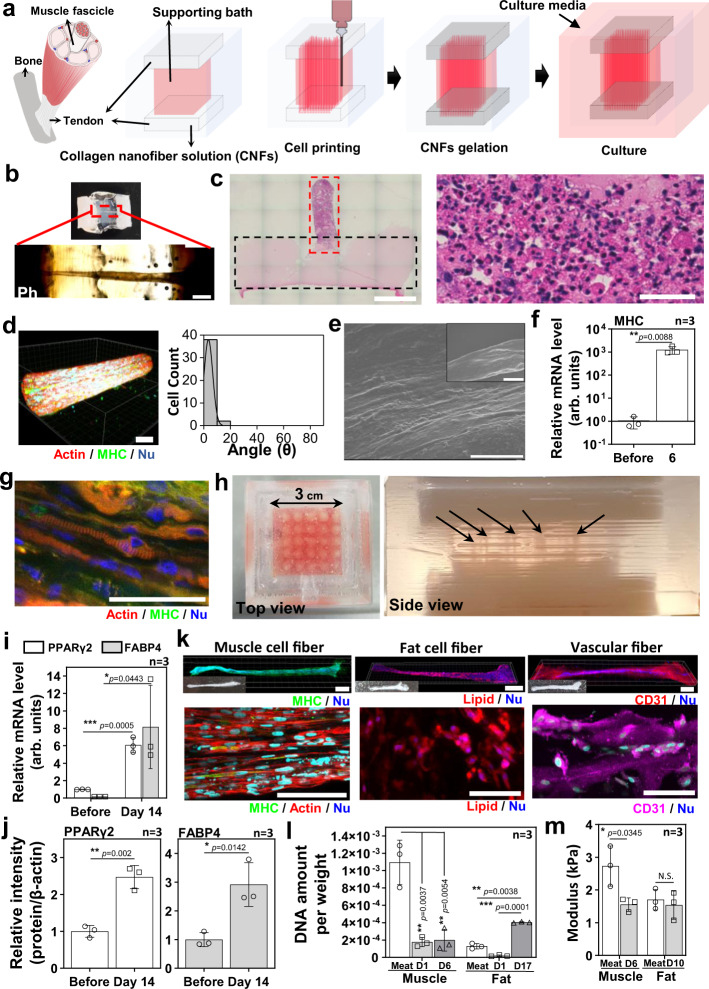
Tendon-gel integrated bioprinting (TIP) for muscle, fat, and vascular tissue fabrication. **a** The schematic of TIP for cell printing. **b** Optical (upper) and phase-contrast (lower) images of the bSC tissue printed by TIP, keeping the fibrous structure on day 3. The images were taken after fixation. Scale bar, 1 mm. **c** The H&E-stained image of half of collagen gel (dotted black line)—fibrous bSC tissue (dotted red line) and a magnified image of the fibrous bSC tissue (right). Scale bars, 2 mm (left) and 50 µm (right). **d** 3D fluorescence image (left) and cell alignment measurement (right) of the TIP-derived bSC tissue stained with actin (red), MHC (green), and nucleus (blue) on day 3 of differentiation. Scale bar, 50 µm. **e** SEM images of TIP-derived bSC tissue on day 3 of differentiation. Scale bars, 10 µm and 100 µm (inset). **f** MHC mRNA expression levels of bSCs before printing and TIP-derived bSC tissue on day 3 of differentiation (*n* = 3 independent samples, pairwise t-test comparison). **g** Fluorescence image of TIP-derived bSCs tissue stained with actin (red), MHC (green), and nucleus (blue) on day 14 of differentiation. Scale bar, 50 µm. **h** The optical images of multiple tissue fabrication (25 ea.) by multiple printing. Black arrows indicate printed cell fibers. **i, j** mRNA levels (**i**) and protein expression levels (**j**) of TIP-derived fat tissues before printing and at day 14 of differentiation (at day 17 of total culture) (*n* = 3 independent samples, pairwise *t*-test comparison). **k** Whole fluorescence (left), optical (inset), and magnified (right) images of muscle (on day 4 of differentiation, green: MHC & blue: nucleus), fat (on day 14 of differentiation, red: lipid and blue: nucleus), and vascular (on day 7, magenta: CD31 and blue: nucleus) tissues fabricated by TIP. Scale bars, 1 mm (left) and 100 µm (right). **l, m** DNA amount per weight (light-gray bars: day 1, and dark-gray bars: day 6 in muscle fiber and day 17 in fat fiber) and (**l**) compressive modulus (**m**) of muscle and fat fibers in the commercial meat (white bar) and TIP-derived (gray bars). The modulus of the muscle fiber on day 3 of differentiation (after 6 days) and the fat fiber on day 7 of differentiation (after 10 days) was measured (*n* = 3 independent samples, paired one-way ANOVA with a Tukey’s HSD post test (l) and pairwise *t*-test comparison (m)). *p<0.05, **p<0.01, ***p<0.001; error bars represent mean ± s.d. Representative images from at least two independent experiments are shown. Source data are provided as a Source Data file.

The important feature in the modified SBP, which we have named tendon-gel-integrated bioprinting (TIP), is the introduction of tendon gels to anchor the printed cell fibers for culture. Figure 4a illustrates the process of the TIP in which the printing bath is divided into three parts: the bottom tendon gel, the supporting bath, and the upper tendon gel. G-Gel is used as a supporting bath as described in the above section and the volume of tendon-gels is filled with 4 wt% collagen nanofiber solution (CNFs) which has a reversible sol-gel transition from 4 °C to 37 °C (Supplementary Fig. 12). To separate the layers and maintain the structure we fabricated polydimethylsiloxane (PDMS) wells (Supplementary Fig. 13). After the bSC fiber gelation inside the PDMS well (Supplementary Movie 3), incubation for 2 h at 37 °C induced the supporting bath and tendon gels to become a solution and a gel, respectively, and the PDMS well was then put in the culture medium.

On day 3, we could confirm that the printed cell fiber maintained its fibrous shape and kept its connection with the two tendon gels, as seen in the phase contrast and the H&E staining images (Figs. 4b and 4c). The cell viability of the bSC fiber by TIP was confirmed up to day 9 (Supplementary Fig. 14). It also showed a high alignment of cells on day 3 of differentiation, which seemed comparable with the needle-fixed culture (Figs. 3e, 4d, and 4e and Supplementary Movie 4). MHC expression of the TIP-derived bSC fiber was relatively lower than in the needle-fixed culture (Supplementary Fig. 15), but the mRNA level of MHC expression was upregulated by >1000-fold on day 3 of differentiation compared with the naive bSCs (Fig. 4f). Interestingly, sarcomere structures testifying a matured state for the muscle fibers were shown in some of the TIP-derived bSC fibers (Fig. 4g), butwe could not show any needle-fixed culture cell fibers after 14 days of differentiation for the comparison. These results imply that the long-term culture in TIP is able to induce a higher muscle-maturation degree compared with the needle-fixed culture. Even though we did not investigate thoroughly here, it may have been caused by the bSC cell adhesion to the collagen gel at anchorage regions for the TIP whereas there is no cell adhesion in the needle-fixed culture (Supplementary Fig. 16). TIP is a promising method for muscle fiber fabrication, but it still has a problem which is the occasional bSC fiber detachment from the tendon gels, especially the bottom tendon gels, during the prolonged cultures due to its strong contraction. Increasing the concentration of the CNFs or using additional cross-linking will hopefully provide a solution to this problem. Moreover, double printing by the addition of another cell fiber by general TIP after rotating the PDMS well 180° following the fabrication of the first one, may be another way of solving the problem. When double printing is performed, the two printed cell fibers close to each other fused into one thicker-cell fiber (Supplementary Fig. 17).

Multiple printing for 25 bSC cell fiber fabrication in one large PDMS well was also performed (Fig. 4h and Supplementary Movie 5). We first aimed to be able to produce directly in one PDMS well a large tissue composed of various types of cell fibers, but we finally fabricated the muscle, fat, and vascular cell fibers individually in this study because each differentiation needed to be induced in a specific medium corresponding to each cell fiber based on the information discussed in the first section. The adipogenesis of the bADSCs-derived fat fiber by TIP was confirmed by the mRNA level and protein expression of PPARγ2 and FABP4 same as in 3D culture. Compared with naive bADSCs, PPARγ2 and FABP4 were upregulated by > 6-fold and > 40-fold respectively in their mRNA expression and >2-fold and >2-fold, respectively, in their protein level on day 14 of differentiation (Fig. 4i and j and Supplementary Fig. 18). Figure 4k, Supplementary Fig. 19, and Supplementary Movies 6–8 show the whole muscle, fat, and blood capillary cell fibers, respectively, independently fabricated by TIP. Even though each cell fiber was fabricated separately, we believe that if a differentiated media for culturing all three types of cell fibers at the same time is developed, the programmed printing of them in desired locations will be feasible.

The characteristics of DNA amount, compressive modulus, and water contents of the muscle and fat cell fibers by TIP were compared with the fibers extracted from a commercial beef (Supplementary Fig. 20). The DNA concentration in the TIP-derived muscle fiber did not change, depending on the culture day while it increased in TIP-derived fat fiber over that of the commercial meat on day 14 of differentiation, implying the proliferation and the significant change in the cell numbers in fat fibers during the culture and the differentiation after printing, which was not the case for the muscle fibers (Fig. 4l). Also, the DNA concentration in the TIP-derived muscle fibers was found six times smaller than the meat fibers (Fig. 4l), indicating that optimization of the bSC concentration or the ECM concentration in the bioink will be necessary to be equivalent to the real meat.

Although the water content showed the disparity between the commercial beef and the TIP-derived cell fibers (Supplementary Fig. 21), while compressive modulus in all cell fibers (muscle fiber on day 3 of differentiation and fat fiber on day 7 of differentiation) showed similar values, which were within one order of kPa (Fig. 4m and Supplementary Fig. 22). Since the TIP-derived cell fibers were not controlled for tenderness, flavor, and additional nutrient components in this study, these factors will need to be addressed to produce customer-oriented cultured meat.

### Engineered steak construction by assembly of muscle, fat, and vascular cell fibers

**Fig. 5:**
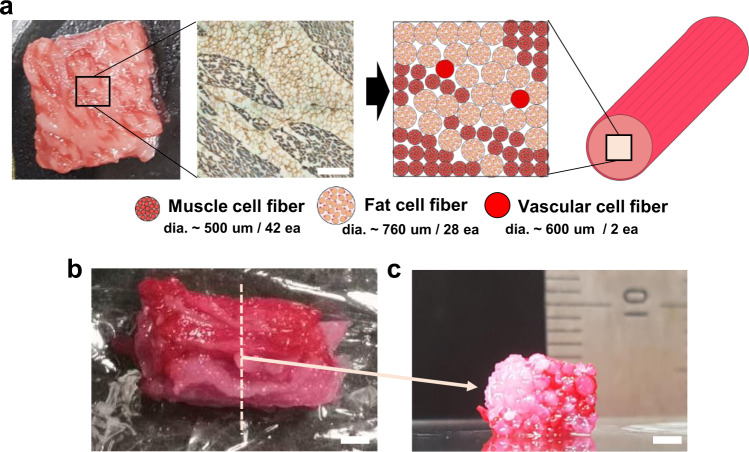
Assembly of fibrous muscle, fat, and vascular tissues to cultured steak. **a** Assembly schematic- (right) based sarcomeric α-actinin (blue) and laminin- (brown) stained image (left) of the commercial meat. It is assumed that the diameters of the fibrous muscle, fat, and vascular tissues are about 500, 760, and 600 µm, respectively. Scale bar, 1 mm. **b, c** Optical images of the cultured steak by assembling muscle (42 ea.), fat (28 ea.), and vascular (2 ea.) tissues at (**b**) the top and (**c**) cross-section view of the dotted-line area. Muscle and vascular tissue were stained with carmine (red color), but fat tissue was not. Scale bars, 2 mm.

The assembly of the TIP-derived cell fibers was attempted to demonstrate the construction of the cultured steak. To mimic the structure of commercial beef, we first took a cross-sectional image of Wagyu with sarcomeric α-actinin and laminin stainings, which denotes the muscle in double-positive and the adipose in laminin-only positive, respectively (Fig. 5a, left). We tried to produce a cultured steak with dimensions of approximately 5 mm × 10 mm × 5 mm (WxLxH) and from the Wagyu’s image, we made the model pattern showing the required number of each muscle, fat, and blood capillary cell fibers as well as their arrangement (Fig. 5a, right). The diameters of the cell fibers obtained by TIP were measured to be approximately 500, 760, and 600 µm, which meant that the required numbers of each cell fiber were 42, 28, and 2, respectively. To distinguish each cell fiber, muscle and vascular cell fibers were stained in red using food coloring, leaving fat cell fiber in white color. After physically stacking the cell fibers like the model image, they were treated with transglutaminase, which is a common food cross-linking enzyme, to accelerate the assembly during two days at 4 °C (Supplementary Fig. 23). The final product is shown in Fig. 5b and a cross-sectional image was taken to verify that the structure was analogous to Wagyu (Fig. 5c), finally implying the feasibility of the TIP-based engineered steak fabrication.

## Discussion

In this research, we reported a technology for constructing a whole cut meat-like tissue with muscle, adipose, and blood capillary cell fibers composed of edible bovine cells. Although cultured meat using livestock cells is drawing huge attention, only few studies give information related to their cellular behavior and the used cells were primary cells^4,7,46^. In our model, after isolation and purification of the bSCs and bADSCs, we first verified their cell behaviors: the proliferation and differentiation of the bSCs (Fig. 2a–c) and the adipogenesis (Fig. 2d–h) and the differentiation to endothelial cells (Fig. 2i–k) from bADSCs, depending on media conditions. Although we used p38 inhibitor to increase the available cell number of bSCs^7^, decreased differentiation capability and proliferation rate of bSCs after passage 8 was shown and it has to be addressed for scalable production of cultured steak in the future. Satellite cells are known to do asymmetric or symmetric cell divisions to regulate cell populations^47^, so an approach based on the division mechanism of satellite cells will be necessary.

The use of ADSCs for their endothelial differentiation allowed us to avoid the direct isolation of bovine endothelial cells, which could have added a limiting step to the full process. Moreover, their differentiation in adipocytes, while previously done, still remains little studied. Especially, the induction of the late marker FABP4 was not reported, while its role in the bovine adipogenesis was indeed found of importance for the bovine lipid metabolism-related gene induction^48,49^. Our study thus provided additional information on both adipogenic and endothelial differentiations from bovine stem cells, which can have other further applications in the meat-related fields.

Furthermore, it was then shown that the resistance to the contraction force during the culture of the bSC-derived cell fiber was essential to realize highly aligned muscle fibrils (Fig. 3c–e). A modified supporting bath-assisted cell printing method, the tendon-gel-integrated printing (TIP), was thus developed, in which the collagen gel-based tendon tissues can withstand the cell traction force during the bSCs differentiation, leading to a well-maintained fibrous structure and its cell alignment (Fig. 4a–f). Comparisons of the cell density (Fig. 4l), compressive modulus (Fig. 4m), and water content (Supplementary Fig. 21) showed the gap between TIP-derived and commercial muscle and fat cell fibers. We demonstrated engineered steak-like tissue, analogous to the structure of commercial beef, through the manual assembly of muscle, adipose, and blood capillary cell fibers produced through the TIP (Fig. 5a–c). Up to our knowledge, this is the first report to demonstrate the fabrication of whole-cut cultured meat-like tissue that was composed of three types of primary bovine cells isolated from an edible meat block and was modeled into a real meat’s structure. Since the demonstrated cultured steak-like tissue is a small piece and inedible, further elaboration will therefore be required with consideration of TIP-based cell printing scalability and edibility of culture and cell-printing-related materials in the future. In addition, to print bovine cells for cultured meat, we expect that the TIP will also benefit the muscle tissue engineering applications in the future.

## Methods

### Isolation and purification of bSCs and bADSCs

bSCs were isolated from 160 g of fresh masseter muscle samples (within 6 h of euthanasia) of 27-month-old Japanese black cattle obtained at the slaughterhouse (Tokyo Shibaura Zouki, Tokyo, Japan, and JA ZEN-NOH Kanagawa, Kanagawa, Japan). The freshly harvested bovine muscle was kept on ice, transferred to a clean bench, and washed with cold 70% ethanol for 1 min, followed by cold PBS 1 x 2 times. Then, the fat tissue part was disposed of, the remaining muscle was cut into small pieces with a knife, and minced with a food processor mechanically. The bovine minced muscle was washed with cold PBS 1x with 1% penicillin–streptomycin (PS) (Lonza, 17-745E) for 1 min. The washed muscle was transferred to a bottle and mixed with 160 ml of 0.2% collagenase II (Worthington, CLS-2) in DMEM (Invitrogen, 41966-29) supplemented with 1% PS. The bottle was incubated and shaken every 10 min for 1.5 h at 37 °C. After digestion, 160 mL of 20% FBS in DMEM supplemented with 1% PS was added and mixed well. The mixed solution was centrifuged for 3 min at 80 g and 4 °C. Floating tissues in the supernatant after centrifugation were removed by tweezers and then the collected supernatant was kept on ice as a mononuclear cell suspension. Precipitated debris were mixed with 80 ml of cold 1% PS in PBS 1x and centrifuged for 3 min at 80 g and 4 °C. The supernatant was collected again and mixed with the former mononuclear cell suspension. After that, the cells were filtered through a 100 μm cell strainer. After centrifugation for 5 min at 1500 g and 4 °C, the cells were suspended with 160 ml of cold DMEM with 20% FBS and 1% PS, were filtered through a 100-μm cell strainer followed by a 40-μm cell strainer. The cells were then centrifuged for 5 min at 1500 g and 4 °C. Precipitated cells were incubated with 8 ml of erythrocyte lysis buffer (ACK, 786-650) for 5 min on ice. Then the cells were washed twice with cold PBS 1x supplemented with 1% PS and the cell pellet was mixed with FBS supplement with 10% dimethyl sulfoxide and then reserved at −150 °C. The frozen cells were recovered in a 37 °C water bath and washed with cold PBS 1x twice. The cells were suspended in F10 medium (Gibco, 31550-023) containing 20% FBS, 5 ng/mL bFGF (R&D, 233-FB-025), and 1% PS supplemented with 10 μM p38i (Selleck, S1076), and then seeded at 1.1 × 10^5^ cells/well in 6-well cell culture plates (Corning) that were coated with 0.05 % bovine collagen type I (Sigma, C4243). The cells were cultured by changing the medium every two or three days and were passaged when they reach 60 % of confluency until passage 3 for cell sorting by flow cytometry. The cells were suspended in FACS buffer (1% BSA in PBS1x) and stained with APC anti-human CD29 Antibody (BioLegend, 303008, TS2/16, dilution 1/40), PE-Cy^TM7^ anti-human CD56 (BD, 335826, NCAM16.2, dilution 1/40), FITC anti-sheep CD31 (BIO-RAD, MCA1097F, CO.3E1D4, dilution 1/40), and FITC anti-sheep CD45 (BIO-RAD, MCA2220F, 1.11.32, dilution 1/40) for 30–45 min on ice in the dark. After antibody incubation, the cells were washed twice with cold PBS 1x and reconstituted in PBS 1x with 2% FBS. The CD31^−^CD45^−^CD56^+^CD29^+^ cells were isolated by Sony Cell Sorter SH800S as bSCs.

Subcutaneous bovine adipose tissues were isolated at the slaughter house on the day of slaughter and sent to the laboratory with an ice pack. Following the 24-h duration delivery, the tissues were first washed in PBS 1x containing 5% of penicillin-streptomycin-amphotericin B (Wako, 161-23181). Then, 8–10 g of tissue was separated into fragments to fill the six wells of a 6-well plate and was minced to get around 1mm^3^ in size using autoclaved scissors and tweezers, directly in 2 mL of collagenase solution containing both collagenase type I (Sigma Aldrich, C0130) and type II (Sigma Aldrich, C6885) at 2 mg/mL for each in DMEM with 1% antibiotics–antimycotic mixed solution (Nacalai, 02892-54), 0% FBS, and 5% BSA (sterilized by 0.2 µm filtration). After one hour of incubation at 37 °C with 250-rpm agitation, DMEM (Nacalai, 08458-16) with 10% FBS and 1% antibiotics was added and the lysate was filtrated using a sterilized 500 µm iron-mesh filter, before being centrifuged for 3 min at 80 g. The upper human mature adipocyte layer was then removed and the pellet containing the stromal vascular fraction (SVF) was washed two times in PBS 1x with 5% BSA and 1% antibiotics and once in complete DMEM (10% FBS + 1% antibiotics), by 3 min of centrifugation at 80 g between each wash. Finally, the pellet containing the SVF cells was resuspended in DMEM and seeded in a 10-cm dish for expansion by changing the medium every day for three days, and the cells were passaged when they reached 80% of confluency. After P1, the remaining adherent cells are considered as bADSCs and were expanded in DMEM.

### 2D cell culture

bSCs were cultured in high-glucose DMEM (Gibco, 10569-010) containing 1% antibiotic–antimycotic mixed solution (Nacalai, 02892-54), 10% FBS, 4 ng/mL fibroblast growth factor (Fujifilm, 067-04031), and 10 uM p38 inhibitor (Selleck, SB203580) for proliferation, and bSCs within P8 were used for all experiments. For differentiation induction of bSCs, the medium was changed to DMEM containing 2% horse serum and 1% antibiotic–antimycotic mixed solution. To investigate the cell proliferation and differentiation ratio, 50,000 cells were seeded on 24-well plates, and the differentiation medium was replaced every two days after differentiation induction.

### Culture and differentiation of bADSCs to bovine endothelial cells

To monitor the endothelial differentiation of bADSCs, bADSCs at P1 previously expanded in DMEM were seeded at 5000 cells on 48-well plates and kept seven days in eight different conditions (DMEM with 1, 5, or 10% HS, DMEM with 10% FBS, DMEM with 10% human serum, DMEM with 10% calf serum, F12K with 10% HS, and F12K with 10% FBS, all containing 1% antibiotics-antimycotic mixed solution) by renewing the medium every 2-3 days.

### Collagen microfiber preparation

The collagen microfibers (CMF) were first prepared from a collagen type I sponge (Nipponham) after dehydration condensation at 200 °C for 24 h crosslinking to prevent its dissolution in water-based solution. The crosslinked collagen sponge was mixed with ultra-pure water at a concentration of 10 mg/mL (pH = 7.4, 25 °C) and homogenized for 6 min at 30,000 rpm. Then, the solution was ultrasonicated (Ultrasonic processor VC50, SONICS) in an ice bath for 100 cycles (one cycle comprised 20 s of ultrasonication and 10 s of cooling), to make smaller fragments that are able to induce better vascularization and filtrated (40-µm filter, microsyringe 25-mm filter holder, Merck), before being freeze-dried for 48 h (FDU-2200, EYELA)^50^. The obtained CMF was kept in a desiccator at RT.

### 3D gel-embedded culture

To construct the adipose tissues by 3D culture, CMF were first weighted and washed in DMEM without FBS by being centrifuged for 1 min at 16,083 g to get a final concentration in the tissues of 1.2 wt%. The bADSCs were added after trypsin detachment (always used at P1–5) and centrifuged for 1 min at 1970 g to get a final cell concentration of 5 × 10^6^ cells/mL. The pellet containing CMF and bADSCs was then mixed in a fibrinogen (Sigma Aldrich, F8630) solution at 6 mg/mL final concentration (the stock solution at 50 mg/mL prepared in DMEM with 1% antibiotics) and the thrombin solution (Sigma Aldrich, T4648) was added to get a final concentration of 3 U/mL (the stock solution at 200 U/mL prepared in DMEM with 10% FBS and 1% antibiotics). Finally, 2 µL drop tissues were seeded in a 96 well plate (Iwaki, 3860-096) and gelated for 15 min in the incubator at 37 °C. Then 300 µL of medium (DMEM with 10% FBS and 1% antibiotics) was added to the drop tissues. For adipogenic differentiation, three days of proliferation were first necessary to allow the bADSC proliferation, until reaching a suitable cell–cell interaction required for the adipogenesis^51^. The medium was then switched for DMEM with 10% FBS containing different adipogenic components to compare: Rosiglitazone (at 20 µM final concentration, Sigma Aldrich, R2408), Insulin (at 10 µg/mL final concentration, Sigma Aldrich, I6634), Troglitazone (at 40 µM final concentration, Sigma Aldrich, T2573), Pristanic acid (at 50 µM final concentration, Funakoshi, 11-1500), Phytanic acid (at 50 µM final concentration, Sigma Aldrich, P4060), Erucic acid (at 50 µM final concentration, Sigma Aldrich, 45629-F), Elaidic acid (at 50 µM final concentration, Sigma Aldrich, 45089), Oleic acid (at 50 µM final concentration, Sigma Aldrich, O1383), Palmitoleic acid (at 50 µM final concentration, Sigma Aldrich, 76169), Myristoleic acid (at 50 µM final concentration, Sigma Aldrich, 41788), TGF type I receptor activin-like kinase 5 inhibitor (ALK5i II, 2-[3-(6-methyl-2-pyridinyl)-1H-pyrazol-4-yl]-1,5-naphthyridine, at 1–10 µM final concentration, Cayman, 14794), or Bovine Endothelial Cell Growth Medium (Cell Applications Inc., B211K-500). The individual seven free fatty acids were compared as well as some other possible different mixtures (pristanic and phytanic acids together, or the other five remaining free fatty acids) following already published possible adipogenesis inducers for bovine ADSCs^29,30^. The 300 µL of differentiation medium was then renewed every 2-3 days.

### Supporting bath-assisted 3D printing (SBP) and culture

G-GG was produced by preparing a 1 wt% gellan gum (Sansho) in PBS 1x, grinding it with a rotor–stator homogenizer for 6 min at 30,000 rpm, centrifuging at 2837 g for 3 min, and removing the supernatant. G-Gel was produced by preparing 4.5 wt% porcine gelatin (Sigma Aldrich, G1890) in DMEM containing 1% antibiotic–antimycotic mixed solution and 10% FBS, putting it at 4 °C overnight for gelation, adding the same volume of DMEM to the gelatin gel, grinding it with a rotor–stator homogenizer for 2 min at 30,000 rpm, centrifuging at 2837 *g* for 3 min, and removing the supernatant. Gellan gum and gelatin were conjugated with fluorescein to measure particle size. After the fabrication of G-GG and G-Gel with fluorescein-conjugated material, the fluorescence images were obtained. Major and minor lengths of 60 particles were measured in G-GG and G-Gel and the particle size was determined by averaging major and minor lengths. The bioink was prepared to be 5 × 10^7^ cells/mL of bSCs in the mixture composed of 20 mg/mL fibrinogen (Sigma Aldrich, F8630) in DMEM and Matrigel (Corning, 356234) (6:4, v/v). The supporting bath was prepared by mixing G-GG or G-Gel with 10 U/mL thrombin (Sigma Aldrich, T4648) before printing. After filling the prepared supporting bath in a glass vial and loading the syringe containing the prepared bioink onto the dispenser instrument (Musashi, Shotmaster 200DS), cell printing was conducted inside the supporting bath maintaining the syringe and bed parts of the instrument at 4 °C. All parts, such as syringes, nozzles, and containers used for cell printing, were sterilized with 70% ethanol and UV treatment. The nozzle gauge, moving speed, and dispensing speed was 16 G, 1 mm/s, and 2 µL/s, respectively. The printed structures inside the supporting baths were incubated inside a sterile cabinet at RT for 1 h to ensure gelation. After gelation, the G-GG was gently removed by pipetting and printed structures were immersed in 50 mM Tris-HCl buffer (pH 7.4) at 37 °C for 30 min, and the same process was repeated one more time. G-Gel was dissolved by incubation at 37 °C for 2 h. The obtained cell fibers after removal of the supporting baths were cultured in the basic medium of bSCs for 2–3 days and then replaced with the differentiation medium. Suspension culture was simply conducted by placing the printed cell fiber on a tissue culture plate, and needle-fixed culture was conducted by fixing both ends of the printed cell fibers onto the silicone rubber with a size of 2 cm × 2 cm × 3 mm (W × D × H) placed on a six-well plate.

### PDMS well fabrication

The parts of PLA molds were fabricated by the FDM 3D printer (Creality, Ender-3) after modeling by Fusion360 and slicing by Cura for the PDMS wells. PDMS (Corning, Sylgard 184) was poured into the assembled PLA mold and cured at 50 °C overnight, the PDMS wells were obtained by removal of the PLA molds.

### TIP and culture

In all, 4 wt% CNFs were produced from collagen sponge (Nipponham, Type I & III mixture) based on the previous method. After cutting a small area of the PDMS wells’ side to make the media flow channel, it was sterilized by 70% ethanol and UV treatments and was then put on a slide glass. The PDMS well was filled with 4 wt% CNFs, G-Gel, and 4 wt% CNFs at the bottom, middle, and top layers, respectively. Cell printing was conducted in the same way as the SBP. After printing cells, the printed area at the top layer of PDMS well was covered one more time with 4 wt% CNFs, incubated at RT for 1 h, then it was incubated at 37 °C for 2 h to dissolve the G-Gel and induce the CNFs gelation, and finally was placed in a culture container. The bioinks were prepared in the same way as in SBP for muscle cell fiber, by mixing 5 × 10^6^ cells/mL of bADSCs in 1.2 wt% CMF and 20 mg/mL fibrinogen solution for fat fibers and 10^7^ cells/mL of endothelial differentiated bADSCs in 1.2 wt% CMF and 20 mg/mL fibrinogen solution for vascular fibers in DMEM.

### Live/dead staining

Printed cell fibers were stained with 2 µM calcein-AM and 4 µM ethidium homodimer-1 (Invitrogen, L3224) in DMEM at 37 °C for 15 min, followed by rinsing with PBS 1x and fluorescence imaging by confocal microscopy (Olympus, FV3000) with 10x or 30x objective lens. The imaging conditions are as below:

*Channel 1 (Live cell); 488* *nm laser (power 0.1* *~* *2%), Ex: 500* *~* *540* *nm, HV:450* *~* *550, gain 1*

*Channel 2 (Dead cell); 561* *nm laser (power 0.1* *~* *6%), Ex: 610* *~* *710* *nm, HV:450* *~* *550, gain 1*

*Channel 3 (Nucleus); 405* *nm laser (power 2* *~* *5%), Ex: 430* *~* *470* *nm, HV:450* *~* *550, gain 1*

To measure the cell viability, at least six images were randomly taken. The image-based cell viability (%) was calculated by dividing the number of non-dead-stained nucleus by the total number of the nucleus in each image and averaging it. The nucleus counting was conducted by using a particle-analysis plugin in ImageJ.

### Histological staining

Tissues were washed once in PBS 1x and then fixed in 4% paraformaldehyde (Wako, 163-20145) overnight at 4 °C, followed by three-time washes in PBS 1x. The samples were then maintained in PBS 1x solution before being mounted in paraffin-embedded blocks. Paraffin-embedded blocks and sections were prepared and hematoxylin/eosin (H/E) stained by the Applied Medical Research Laboratory, Inc. Some pieces of commercial (Wagyu) beef steak were immersed in 4% paraformaldehyde solution overnight at 4 °C, and then in 1/15 M phosphate buffer (pH 7.4) containing 30% sucrose. They were rapidly frozen in dry ice acetone and cut into 20 µm thick sections. The tissue sections were processed for immunostaining for sarcomeric alpha-actinin and laminin to depict myotubes of skeletal muscles and basement membranes of the muscles and adipose tissues. The immunostaining was performed by use of specific antibodies against sarcomeric alpha actinin (abcam, ab9465, EA-53, dilution 1/1000) and laminin (Sigma-Aldrich, L9393, polyclonal, dilution 1/100), and the reaction products were visualized in blue (Vector Laboratories, Vector Blue) and brown (DAKO, DAB+ chromogen), respectively.

### Immunostaining

Immunostaining was conducted by a general process, 4% paraformaldehyde fixation at RT for 15 min or at 4 °C overnight, permeabilization with 0.2% Triton X-100 (Sigma Aldrich, T8787) at RT for 15 min, blocking with 1% BSA (Sigma-Aldrich, A3294) for 30–60 min, incubation with the 1^st^ antibody in 1% BSA at RT for 2 h or at 4 °C overnight, incubation with cocktails containing fluorophore-conjugated 2^nd^ antibody and 1 µg/mL TRITC-phalloidin (Sigma Aldrich, P-1951) for actin staining or 100 ng/mL NileRed (TCI, N0659) for lipid staining at RT for 1 h, and finally 300 nM DAPI (Invitrogen, D21490) counterstaining. Myosin 4 Monoclonal Antibody (eBioscience, 14-6503-82, MF20, dilution 1/500) for bSCs, Anti-CD31 (Wako, M0823, JC70A, dilution 1/100) for bovine endothelial cells, and PPAR gamma (Abcam, ab45036, polyclonal, dilution 1/100) and FABP4 (LSBio, LS–B4227, polyclonal, dilution 1/100) antibodies for bovine adipocytes were used as 1^st^ antibodies. Goat anti-Mouse IgG (H+L) Cross-Adsorbed Secondary Antibody, Alexa Fluor 488 (Invitrogen, A-11001, polyclonal, dilution 1/200) and goat anti-Mouse IgG (H+L) Cross-Adsorbed Secondary Antibody, Alexa Fluor 647 (Invitrogen, A-21235, polyclonal, dilution 1/200) were used as 2^nd^ antibodies. All fluorescence images were taken by confocal microscopy. In the case of printed cell fibers, they were treated with Rapiclear 1.52 (SUNJin Lab) at RT for 30 min for deep-tissue imaging.

### Rheology measurement

Viscosity was measured by the controlled-rate mode of the rheometer (Thermo Scientific, HAAKE RheoStress 6000) to verify the thixotropy of G-Gel and G-GG. The steps were composed at 0.01/s for 30 s, at 30/s for 100 s, and at 0.01/s for 30 s.

### UV–Vis

The disposable cuvette was filled with 1 mL of 4 wt% CNFs, and then transmittance was measured at the temperature-controlled steps by UV–Vis spectrometer (Jasco, V-670) at 4 °C for 2000 s, at 37 °C for 2000 s, at 4 °C for 2000 s, and then at 37 °C for 2000 s. After each temperature change, the photos of the samples were taken.

### Mechanical test

The elastic modulus of these printed fibers was measured with EZ test (SHIMADZU, EZ/CE 500 N). All fibers, including printed cell fiber and fibrous tissues from commercial meat, were fixed by 4% paraformaldehyde and washed several times before measurement. After preparing the different printed fibers, several fibers were stacked on a 24-well insert for the sample surface to become larger than the geometry’s surface area at RT. Spherical mold (5 mm in diameter) was used to measure the elastic modulus at a head-moving speed of 1.0 mm/min. The compressive test protocol was employed to increase the engineering strain, until the testing stress to 200 mN. The modulus is automatically calculated by EZ test in the elastic range (10–20 mN). The total sample size was *n* = 3 for each fiber type.

### Water-content measurement

The water content is calculated according to the mass before and after freeze-drying. Briefly, the printed fibers in PBS1x were taken out, the surface liquid was removed, and the wet weight (W_wet_) of the fibers was measured by a balance. The dry weight (W_dry_) of the fibers was measured after freeze-drying (24 h). The water content is given by the following formula (1):

1 $${V}_{\rm {water}}=\frac{{{{{{{\rm{W}}}}}}}_{\rm {wet}}-{{{{{{\rm{W}}}}}}}_{\rm {dry}}}{{{{{{{\rm{W}}}}}}}_{\rm {wet}}}\times 100$$

### DNA content measurement

Commercial beef was bought from the supermarket and intramuscular fat tissues as wells as muscle tissue parts were cut into small fibers of the same size as the printed fibers (Fig. S19). One fiber from the commercial fibers or printed fibers was put per microcentrifuge tube and the tissues were lysed following the DNeasy Blood & Tissue Kit (QIAGEN, 69504) to extract their DNA content, which was quantified by the NanodropTM N1000 device (Thermo Fisher Scientific). Then the DNA amount was presented normalized by the weight of the samples before lysis.

### Bovine-cell fiber assembly

Each cell fiber (muscle, fat, and vascular fibers) was stacked on a plastic container one by one according to the model image obtained from the commercial meat’s histological image. After dispensing transglutaminase solution (10 U/mL, Ajinomoto) onto the stacked cell fibers, they were wrapped up and kept at 4 °C for two days. To take a cross-sectional picture, we cut it and pill off the plastic wrap.

### Image processing and analysis

For the evaluation of bSCs differentiation on 2D culture, at least 4 fluorescence images of the samples stained with DAPI and MF20 were taken in size of about 6 mm × 6 mm, and the number of nuclei in all areas and MHC-positive area was measured by ImageJ. For the measurement of cell alignment degree, we randomly selected the fluorescence images (actin stained with TRITC-phalloidin) after Z-stack imaging of one printed tissue, chose three 2D images showing clear-cell morphologies, and measured the angle of individual cells to printed cell fiber’s major axis by using ImageJ. The number of measured cells in each condition was in the range of 27–56. For the calculation of lipid production from bADSCs, the 3D tissues’ Z-stack images were taken (3 slices with 50 µm step) with the same exposure time, brightness, and contrast, then the summation of Z-slice’s intensity in Nile Red and Hoechst of each 3D tissue was done by Image J. The relative intensity was calculated from the total Nile Red’s intensity divided by the Hoechst intensity in each 3D tissue.

The 4x lens (0.16 dry, WD 13.0) of the confocal quantitative image cytometer CQ1 (Yokogawa, Tokyo, Japan). was used, with the following parameters:

Channel 1 (Nile Red); 561-nm laser (power 20%), Ex: 617~673 nm, Exposure time 500 ms, Bin 1, Gain 16-bit, low noise, and high well capacity, contrast enhancement maintained constant at 100–3200. Channel 2 (Hoechst); 405-nm laser (power 100%), Ex: 447~460 nm, Exposure time 500 ms, Bin 1, Gain 16-bit, low noise, and high well capacity, contrast enhancement maintained constant at 320–1300.

For the differentiation from bADSCs to endothelial cells, the same method was applied with 2D images taken by the CQ1 confocal, using CD31’s total fluorescence intensity normalized by Hoechst total fluorescence-intensity ratios, at the same parameters for all conditions compared. The 3D-reconstructed image and the printed cell fibers’ 3D movie was obtained by Imaris software (Bitplane).

### Gene expression

Gene expression was analyzed using real-time quantitative polymerase chain reaction (RT-qPCR). Adipose drop tissues at days 0, 7, and 14 of differentiation, as well as bioprinted fiber samples at days (before bioprinting) and at days 6 (myoblast fibers), 7 (endothelial fibers), or 14 (adipocyte fibers) were washed in PBS and total RNA extraction was carried out using the PureLink RNA Micro Kit (Invitrogen, Waltham, USA), with the DNAse step, following the manufacturer’s instructions. Samples’ RNA content was quantified with the NanodropTM spectrometer (N1000, Thermo Fisher Scientific, Waltham, USA). For RT-qPCR, the RNA samples were first submitted to reverse transcription into cDNA using iSCRIPT cDNA synthesis kit (Bio-Rad, Hercules, USA), before being amplified using Taqman probes and reagents (Taqman Fast Advanced Mix, Taqman gene expression assays (FAM): *MYH2* (Assay ID: Bt03223147_gH), *FABP4* (Assay ID: Bt03213820_m1), *CD31/PECAM1* (Assay ID: Bt03215106_m1), *PPARG* (Assay ID: Bt03217547_m1), and *PPIA* (Assay ID: Bt03224615_g1), Thermo Fisher Scientific, Waltham, USA). The cDNA synthesis and RT-qPCR reactions were conducted using the StepOnePlus Real-Time PCR System (Thermo Fisher Scientific, Waltham, USA) and the gene expression was normalized by *PPIA* as the housekeeping gene.

### Western blot

Proteins relative expression was assessed by performing western blot. Myoblast (days 0 and 6), endothelial (days 0 and 7), and adipocyte (days 0 and 14 of differentiation) fibers were washed with PBS and homogenized by pipetting in lysis buffer (RIPA Buffer R0278, Sigma-Aldrich with Protease Inhibitor Cocktail 25955-24, Nacalai-Tesque), before being quantified by a BCA Protein Assay Kit (23225, ThermoFisher Scientific). About 4–10 μg of protein was resolved on each lane of 4–15% protein gels (Mini-PROTEAN^®^ TGX Stain-Free™ 4568084, BIORAD with Running Buffer for SDS-Tris-Glycine (10X), Cosmobio), electrotransferred onto a PVDF membrane (Immun-Blot^®^ 1620-0176 BIORAD), and probed using specific antibodies: Myosin 4 Monoclonal antibody (eBioscience, 14-6503-82, dilution 1/1000), PPAR gamma antibody (Abcam, ab45036, polyclonal, dilution 1/1000), FABP4 antibody (LSBio, LS–B4227, polyclonal, dilution 1/1000), and β-Actin antibody (Sigma-Aldrich, A5441, AC-15, dilution 1/3000). Proteins were detected by secondary antibodies conjugated to horseradish peroxidase (Anti-Mouse IgG 170-6516, anti-Rabbit IgG 170-6515, dilution 1/5000, and Precision Protein^TM^ StrepTactin-HRP Conjugate 161-0380, BIORAD, dilution 1/5000). Signal detection was performed by an enhanced chemiluminescence-detection reagent (Clarity Western ECL Substrate 1705060, BIORAD) using the ChemiDoc Imaging System (BIORAD). Molecular weights were determined by comparison with the migration of prestained protein standards (Precision Plus ProteinTM KaleidoscopeTM Standards 161-0395, BIORAD). Quantitative estimation of the bands’ intensity was performed using ImageJ software.

### Statistical analysis

Statistical analysis was performed using EzAnova (version 0.98, University of South Carolina, Columbia, SC, USA) and GraphPad Prism 8 (version 8.4.3 (686), San Diego, USA) software. The detail of the number of n corresponding to the number of independent experiments using isolated bovine primary cells from different bovine donors, or independent samples, is displayed on each graph in the figures and in the captions, as well as the exact *p* values. For paired samples when the same cells were measured at different times (Fig. 2f and h), a one-way ANOVA with a repeated measures design was used, with the Greenhouse–Geisser correction and the Tukey’s HSD post test for the multiple comparisons. When they were subjected to different treatments (Fig. 2e, I, 4 l, Supplementary Fig. 3, and Supplementary Fig. 15) a classic one-way ANOVA was used, with the Brown-Forsythe and the Bartlett’s tests as well as the Tukey’s HSD post-test for the multiple comparisons. When time and treatments were both involved (Fig. 2d), a two-way ANOVA was applied with time set as “paired or repeated measured” and the treatment as classic analysis or “unpaired”, which led to a pairwise comparison, with the Greenhouse–Geisser and Huynh–Feldt corrections as well as the Tukey’s HSD post test for the multiple comparisons. When two different treatments were applied to the cells (Fig. 2j), a classic two-ways ANOVA was performed, with the Šidák post test for the multiple comparisons. ANOVA was used when more than two conditions were compared with each other and for only two conditions, the pairwise *t*-test comparison was performed (Fig. 4f, i, j, m, Supplementary Fig. 17, and Supplementary Fig. 21). EzAnova software was used only for Fig. 2d analysis, for the other statistical analysis, GraphPad Prism software was used. Error bars represent SD. *p* values were considered significantly different at least when *p* < 0.05. When no marks are shown on the graphs, it means that the differences are not significant.

### Reporting summary

Further information on research design is available in the Nature Research Reporting Summary linked to this article.

## Supplementary information

Supplementary Information

Description of Additional Supplementary Files

Supplementary Movie 1

Supplementary Movie 2

Supplementary Movie 3

Supplementary Movie 4

Supplementary Movie 5

Supplementary Movie 6

Supplementary Movie 7

Supplementary Movie 8

Reporting Summary

## Data Availability

All relevant data supporting the key findings of this study are available within the article and its Supplementary Information files or from the corresponding author upon reasonable request. Source data are provided with this paper.

## Source data

Source data
