# Supplementary material for: Engineered whole cut meat-like tissue by the assembly of cell fibers using tendon-gel integrated bioprinting
Source: Nat Commun. 2021 Aug 24;12:5059. doi: 10.1038/s41467-021-25236-9 (PMC8385070; doi:10.1038/s41467-021-25236-9)
Supplement: Supplementary file 1 — Supplementary Information [file 41467_2021_25236_MOESM1_ESM.pdf]

Engineered Whole Cut Meat-like Tissue by the Assembly of Cell Fibers using Tendon-Gel Integrated Bioprinting

*Dong-Hee Kang[1], Fiona Louis[2], Hao Liu[1], Hiroshi Shimoda[3], Yasutaka Nishiyama[4], Hajime Nozawa[5], Makoto Kakitani[5], Daisuke Takagi[6], Daijiro Kasa[7], Eiji Nagamori[8], Shinji Irie[2,9], Shiro Kitano[2,9], and Michiya Matsusaki[1,2]**

[1]Division of Applied Chemistry, Graduate School of Engineering, Osaka University, 2-1 Yamadaoka, Suita, Osaka 565-0871, Japan

[2]Joint Research Laboratory (TOPPAN INC.) for Advanced Cell Regulatory Chemistry, Graduate School of Engineering, Osaka University, 2-1 Yamadaoka, Suita, Osaka 565-0871, Japan

[3]Department of Anatomical Science, Hirosaki University Graduate School of Medicine, 5 Zaifu-cho, Hirosaki, 036-8562, Japan.

[4]NH Foods, Ltd., 3-3 Midorigahara, Tsukuba, Ibaraki 300-2646, Japan.

[5]Kirin Central Research Institute, Kirin Holdings Company, Ltd., 26-1, Muraoka-Higashi 2, Fujisawa Kanagawa 251-8555, Japan.

[6]Biomedical Business Center, Healthcare Business Group, Ricoh Company, Ltd., 3-25-22 Tonomachi LIC 322, Kawasaki-shi, Kanagawa 210-0821, Japan.

[7]Solution Planning, Product Solution Technologies, Production Printing, Industrial Solutions, Ricoh Japan Corporation, 4-2-8 Shibaura, Minato-ku, Tokyo 108-0023, Japan.

[8]Department of Biomedical Engineering, Faculty of Engineering, Osaka Institute of Technology, 5-16-1 Omiya, Asahi-ku, Osaka 535-8585, Japan

[9]TOPPAN INC., Technical Research Institute, 4-2-3 Takanodaiminami, Sugito-machi, Saitama, 345-8508, Japan.

*Corresponding author foot note:

Professor Michiya Matsusaki, PhD

E-mail: m-matsus@chem.eng.osaka-u.ac.jp

Keywords: Engineered steak-like meat, Tendon-integrated bioprinting, Cell fibers

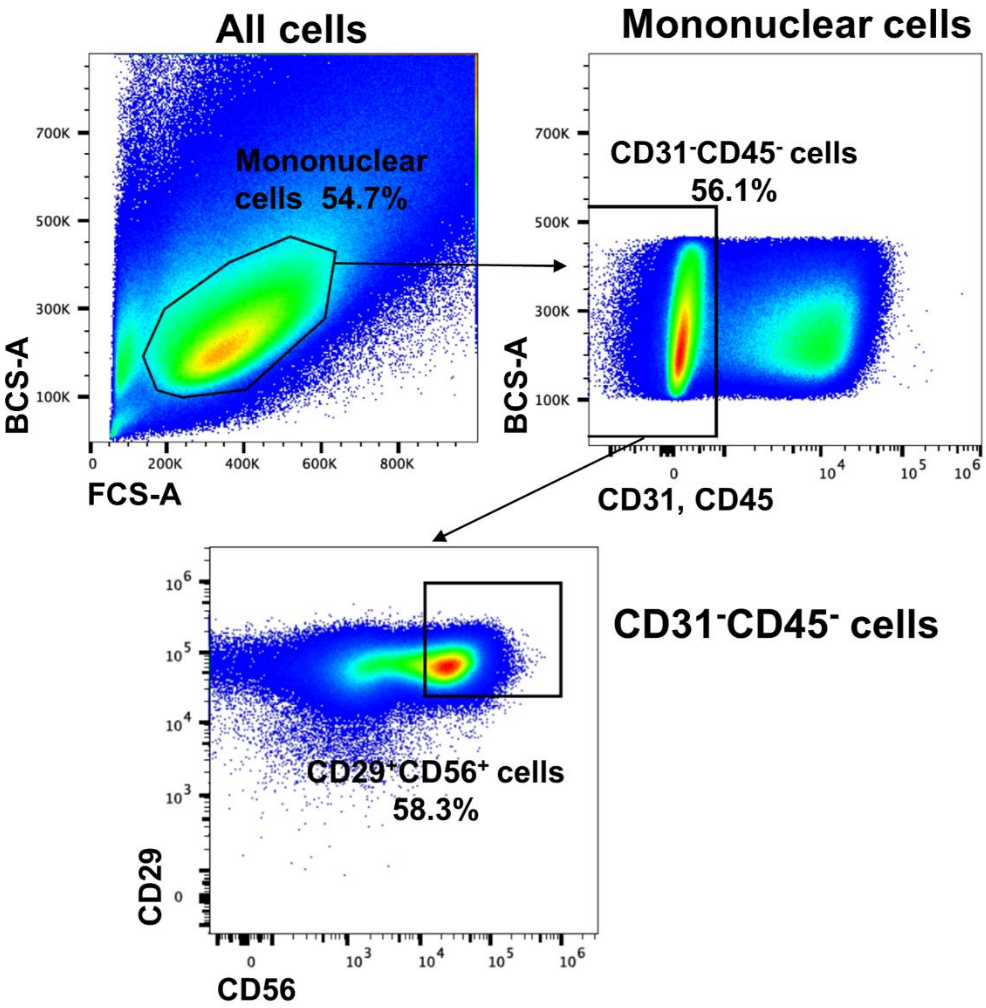

Supplementary Fig. 1 Bovine satellite cells isolation by fluorescence-activated cell sorting. Gating strategy for cell sorting of bovine CD31-CD45-CD29+CD56+ cells.

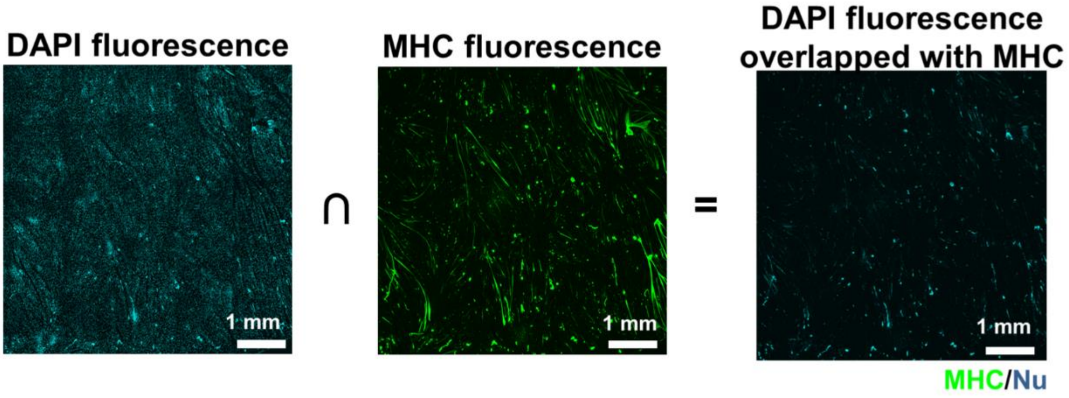

Supplementary Fig. 2 Measurement method of differentiation ratio of bSCs into muscle cells. 'Whole DAPI fluorescence' is divided by 'DAPI fluorescence overlapped with MHC'. DAPI fluorescence overlapped with MHC is obtained in ImageJ software (green: MHC & blue: nucleus). Representative images from three independent experiments are shown.

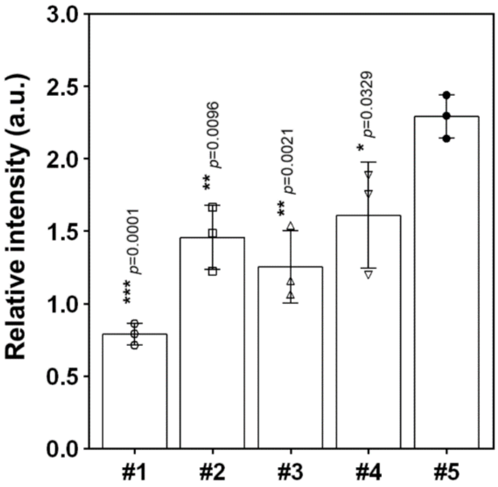

**#1**	**DMEM + insulin 10μg/mL**
**#2**	**DMEM + insulin 10μg/mL + troglitazone 40μM**
**#3**	**Bovine endothelial medium + insulin 10μg/mL**
**#4**	**Bovine endothelial medium + insulin 10μg/mL + troglitazone 40μM**
**#5**	**DMEM + the 7 FFA**

Supplementary Fig. 3 Comparison of free fatty acids and conventional adipogenesis conditions. Adipogenesis ratio (left) of 3D gel drop cultured bADSCs derived by several conditions (right) on day 7. The used bADSCs were extracted from a subcutaneous fat (n = 3 independent samples, unpaired one-way ANOVA with a Tukey's HSD post-test compared to the condition #5). *p<0.05, **p<0.01, ***p<0.001; error bars represent mean ± s.d. Source data are provided as a Source Data file.

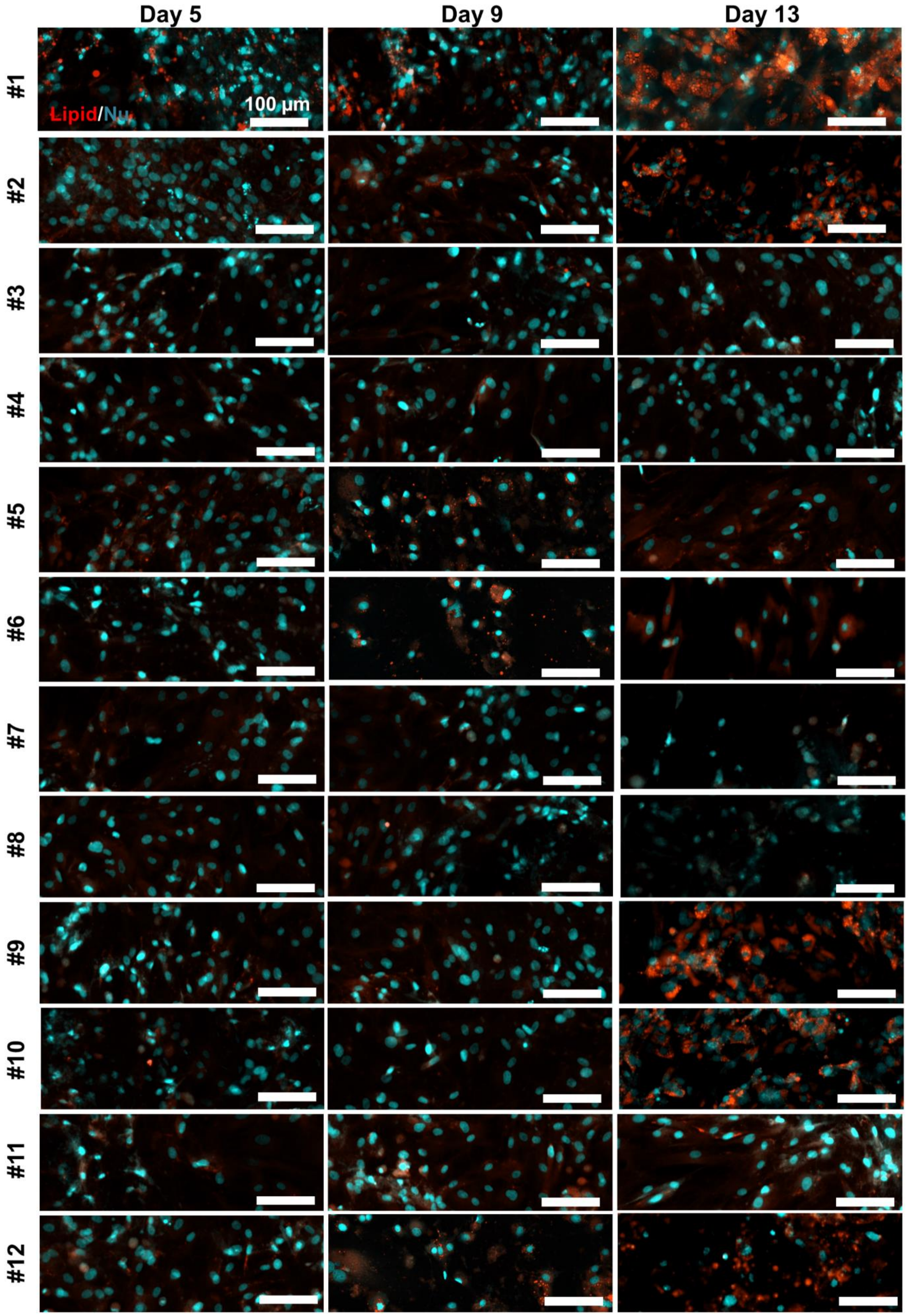
Day 5
Day 9
Day 13
#1
#2
#3
#4
#5
#6
#7
#8
#9
#10
#11
#12
Lipid/Nu
100 μm

Supplementary Fig. 4 Summed z-stack projections immunofluorescence images of the lipid droplet production of bADSCs by 12 combinations of free fatty acids, or rosiglitazone (#4), in DMEM. The used bADSCs were extracted from subcutaneous fat and all images were taken on day 5, 9, and 13 (red: lipid & blue: nucleus). Representative images from four independent experiments are shown.

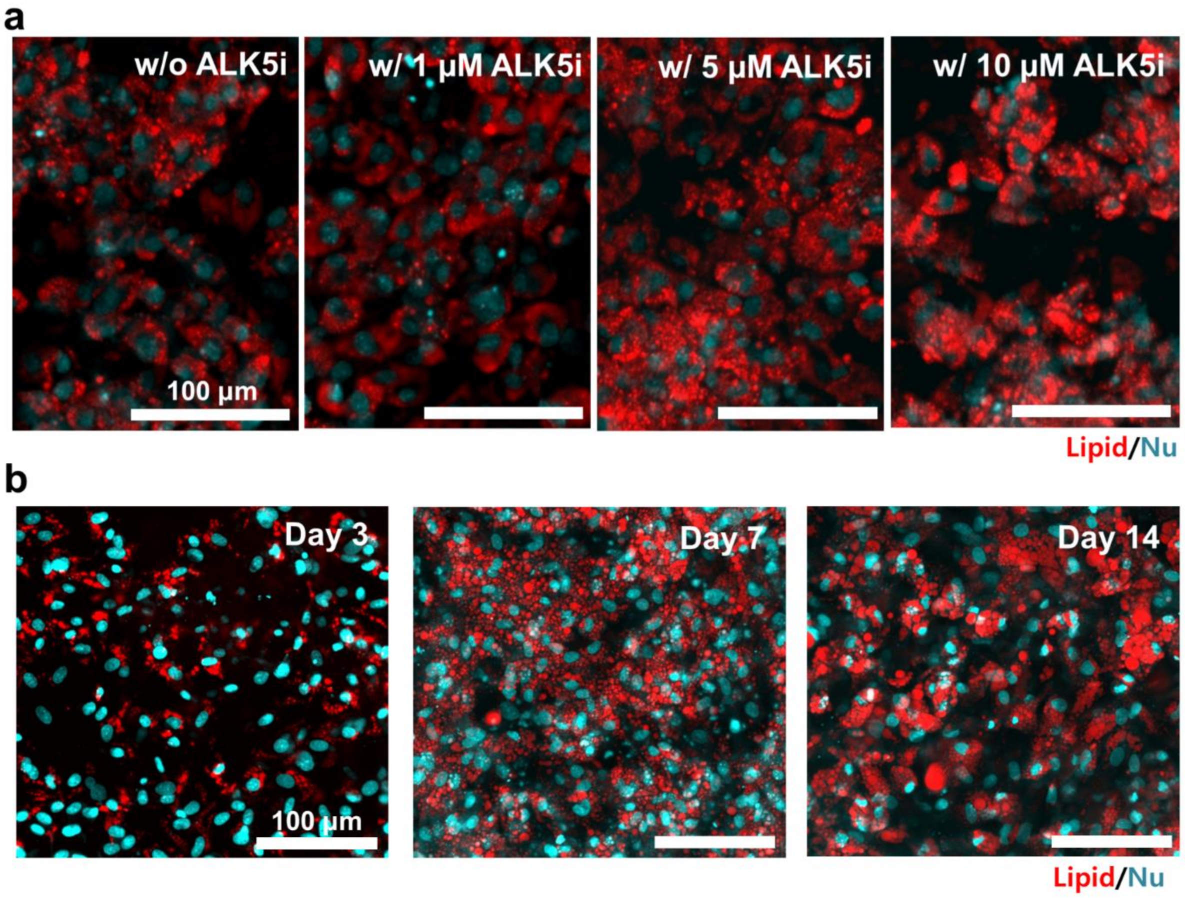

Supplementary Fig. 5 Summed z-stack projections immunofluorescence images of 3D cultured bADSCs depending on the concentration of ALK5i and culture day. (a) With different concentrations (0, 1 μM, 5 μM, and 10 μM) of ALK5i at day 7 and (b) different culture day (3, 7, and 14) in the #1 combination of free fatty acids (red: lipid & blue: nucleus). Representative images from five (a) and three (b) independent experiments are shown.

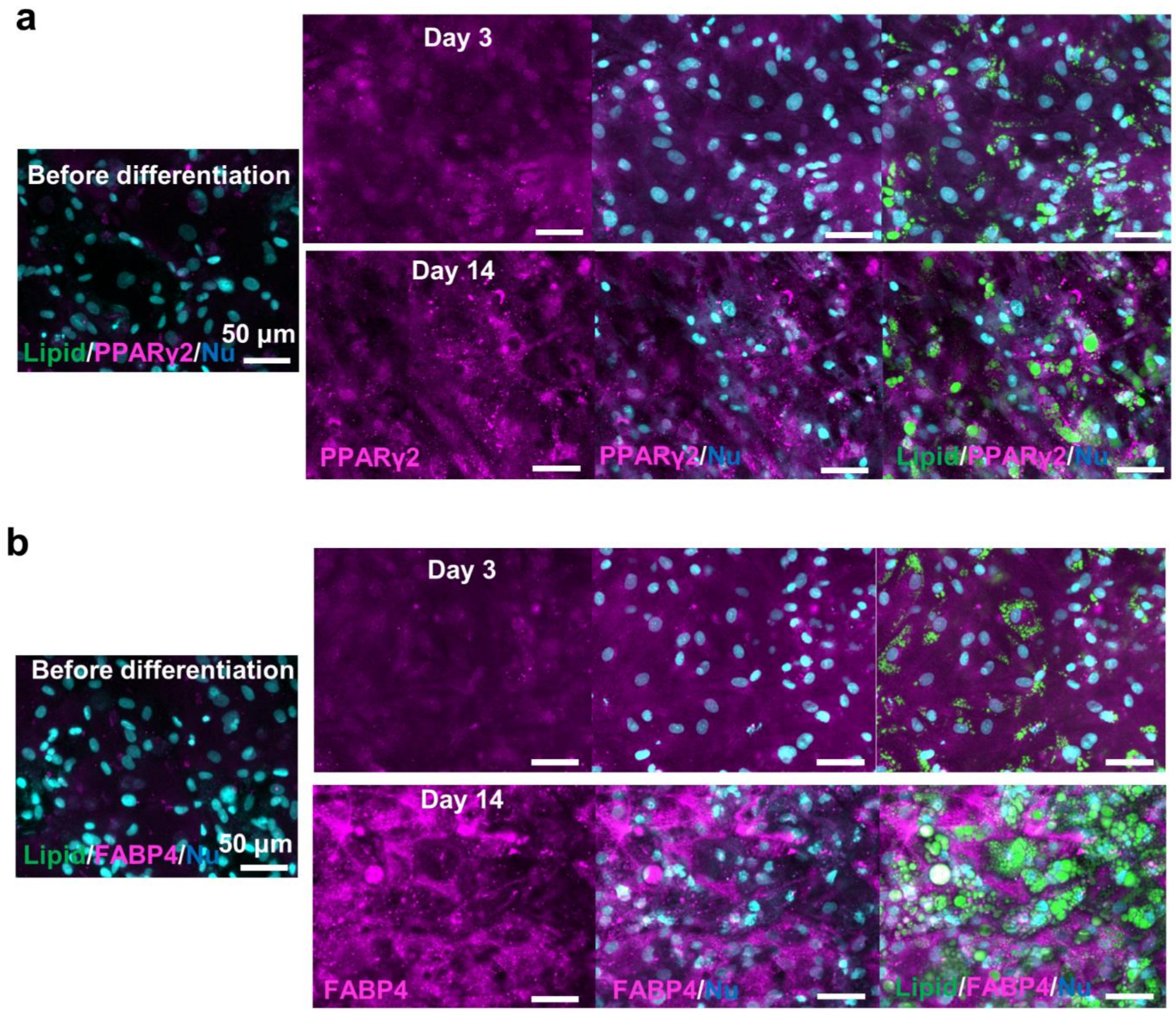

Supplementary Fig. 6 Z-stack projections immunofluorescence images of 3D cultured bADSCs in the optimized media condition. Immunostaining images of **(a)** PPARγ2 and **(b)** FABP4 at before differentiation, day 3, and day 14 (magenta: PPARγ2 or FABP4, green: lipid, and blue: nucleus). Representative images from three independent experiments are shown.

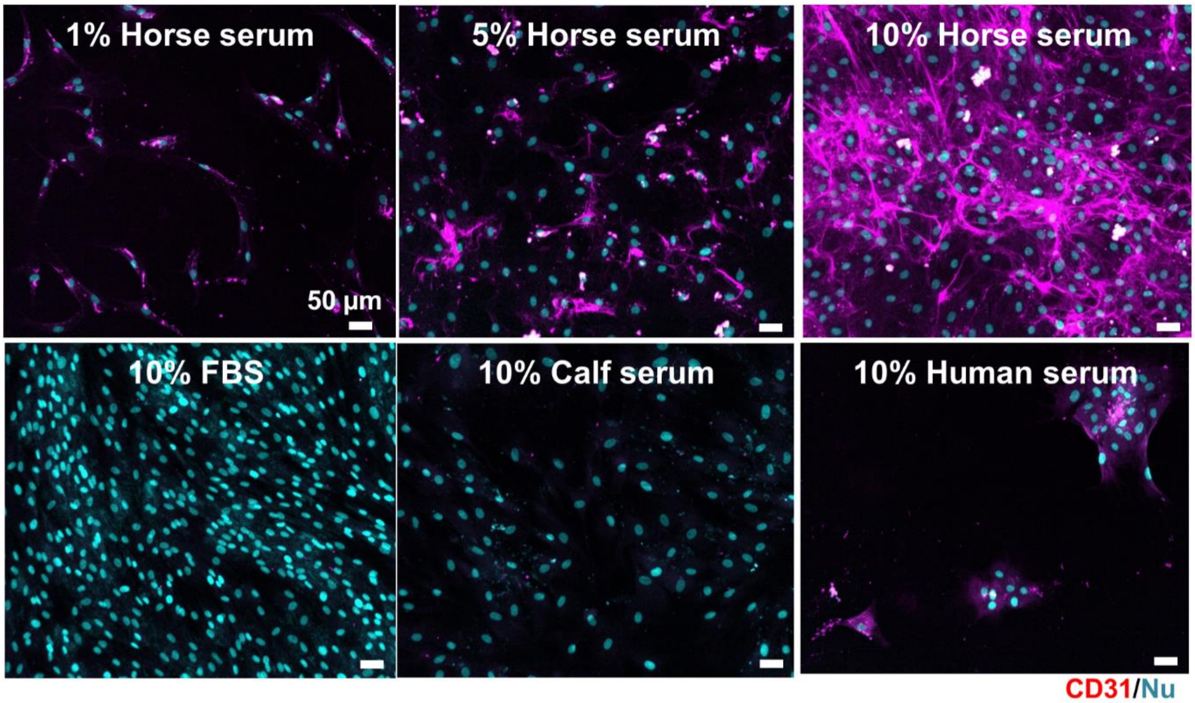

Supplementary Fig. 7 Immunofluorescence images of CD31 expression of bADSCs depending on serum conditions. The used bADSCs were extracted from a subcutaneous fat tissue and cells were stained with CD31 (magenta) and nucleus (blue) on day 7. Representative images from three independent experiments are shown.

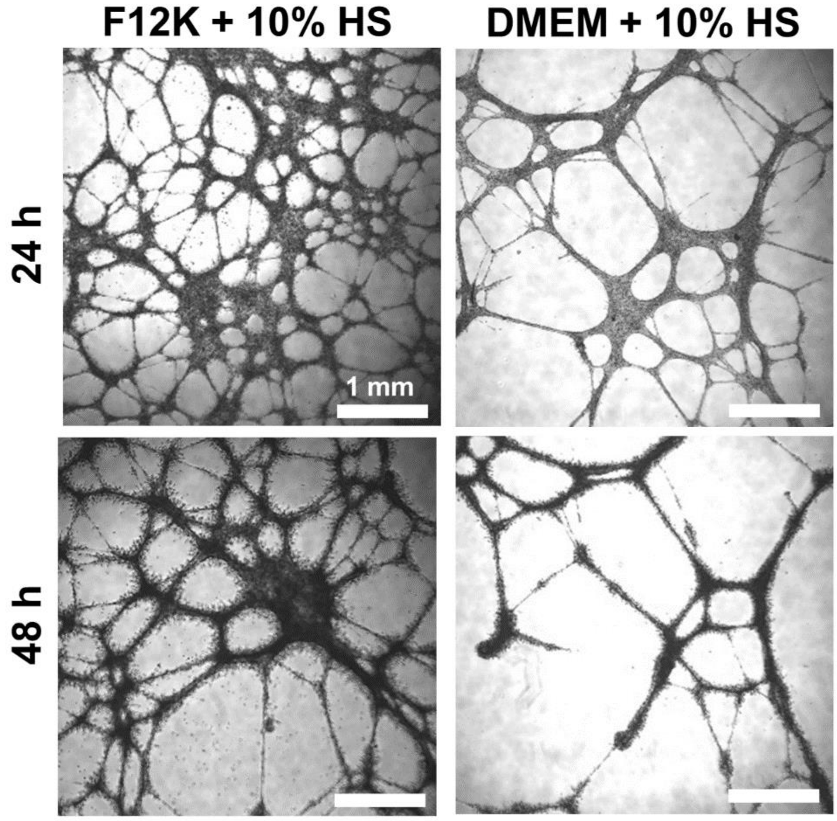

Supplementary Fig. 8 Tubulogenesis assay. Bright-field images of bADSCs (5x10^5 cells/well) that were cultured on Matrigel (10mg/mL, 300µL in a 24 well plate) in F12K (left) and DMEM (right) with 10% HS at 24 h and 48 h. Representative images from one experiment are shown.

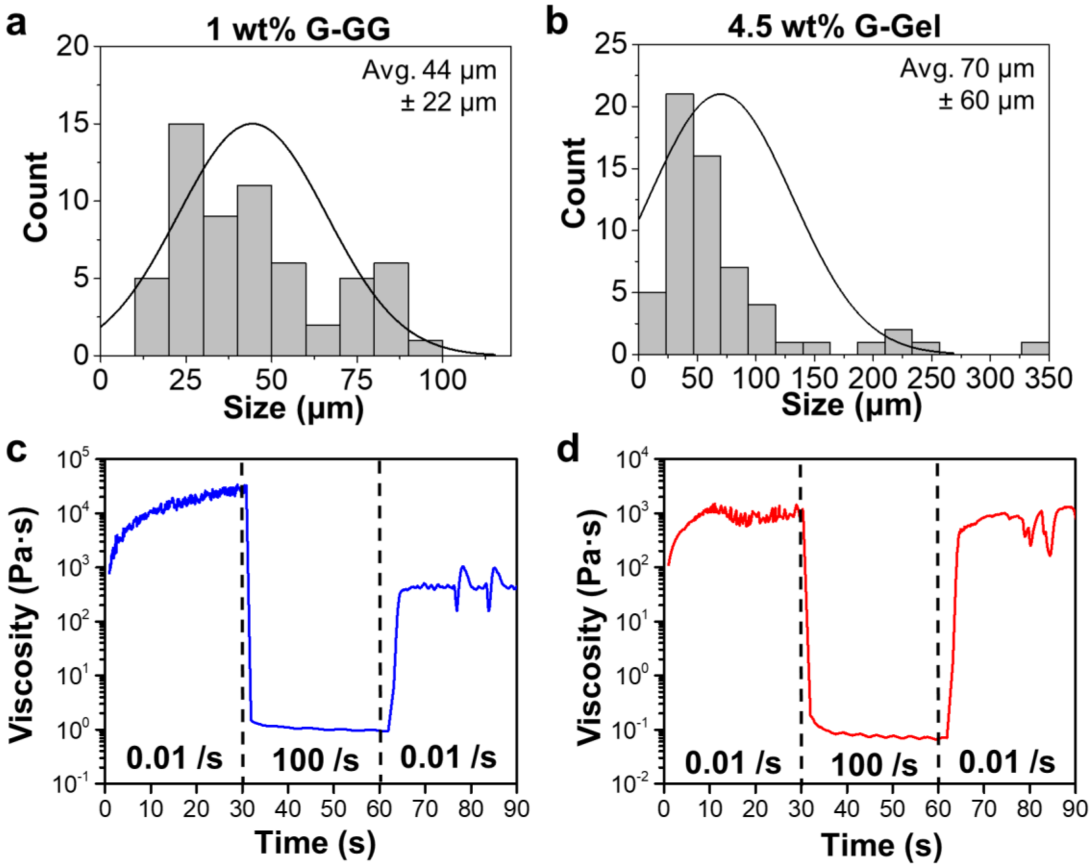

Supplementary Fig. 9 Particle size distributions and rheological measurements of G-GG (a & c) & G-Gel (b & d). Viscosity was measured in 3 steps of controlled shear rate mode; low-shear rate (0.01 /s) for 30 s, high-shear rate (100 /s) for 30 s, and low-shear rate (0.01 /s) for 30s. Source data are provided as a Source Data file.

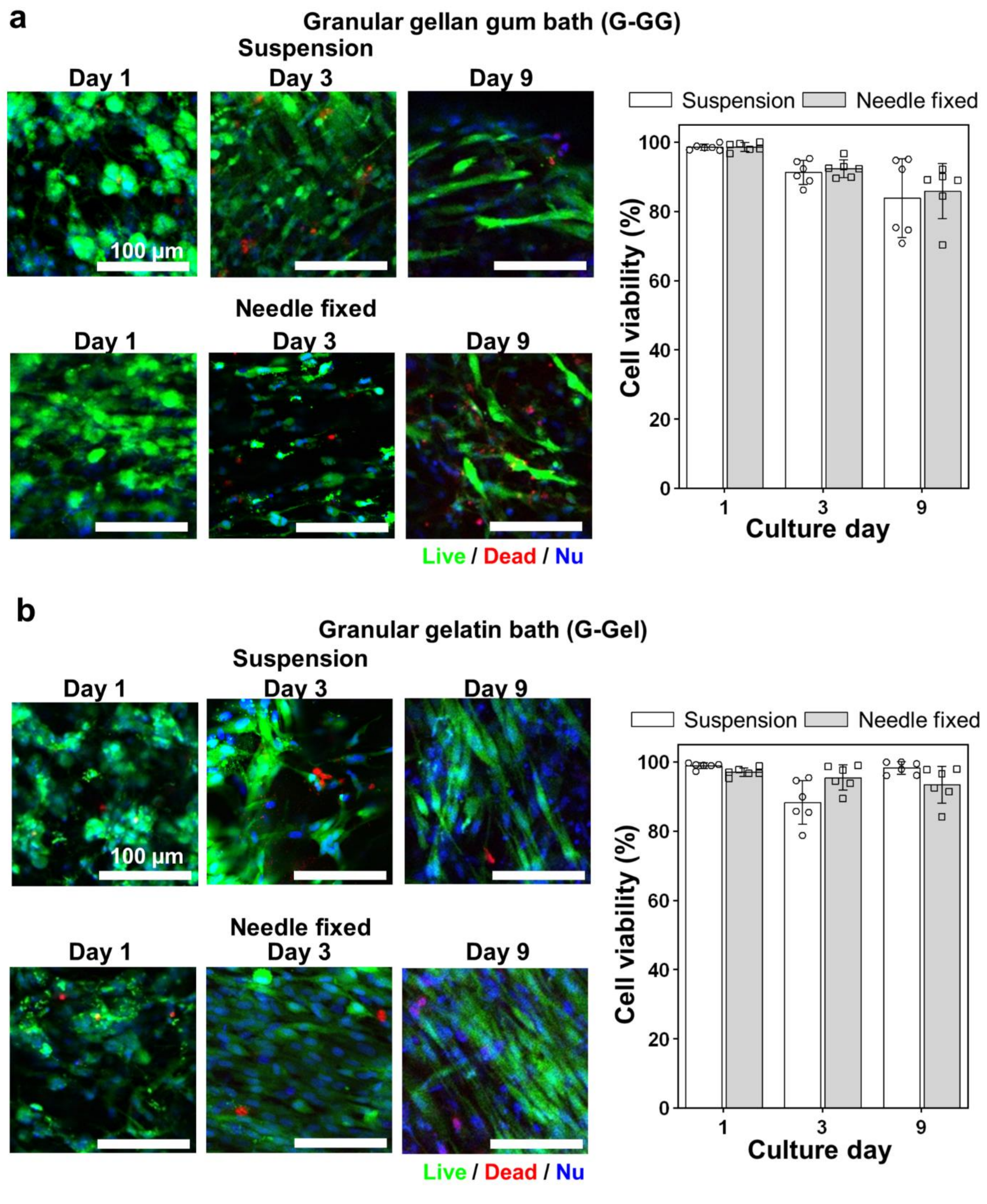

Supplementary Fig. 10 Cell viability of bSCs by SBP. Representative images (left) and the image-based cell viability (right) at day 1, 3, and 9 after printing inside granular gellan gum bath (a) and granular gelatin bath (b) (n = 6 independent areas examined over independent 2 or 3 samples, green: live cells, red: dead cells, and blue: nucleus). Error bars represents mean ± s.d. Source data are provided as a Source Data file.

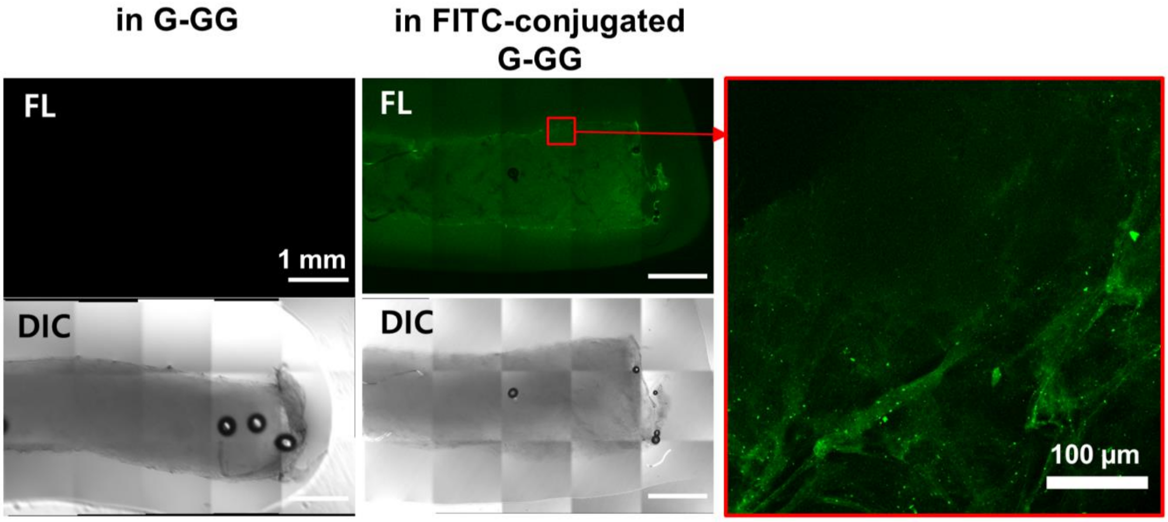

Supplementary Fig. 11 Remaining G-GG in printed structure. The DIC images & fluorescence (FL) images of fibrin gels printed inside G-GG (left) and FITC-conjugated G-GG (right) bath after the removal process in which the printed fiber was immersed at 50 mM Tris-HCl buffer (pH 7.4) for 1h. The buffer was replaced with a fresh one every 30 min. Representative images from two independent experiments are shown.

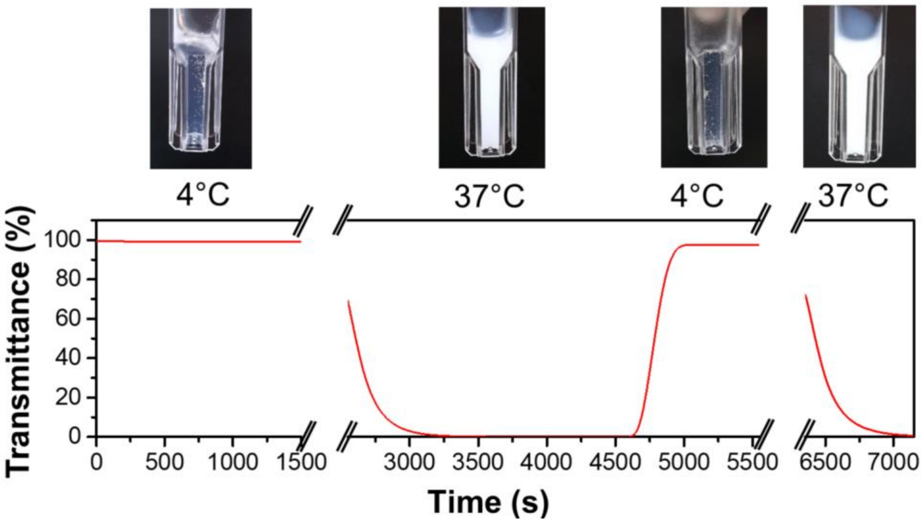

Supplementary Fig. 12 Sol-gel behavior of collagen nanofiber solution (CNFs). The optical images (upper) and transmittance (lower) of 4 wt% CNF fabricated from the mixture of collagen type I & III at steps of 4℃, 37℃, 4℃, and 37℃.

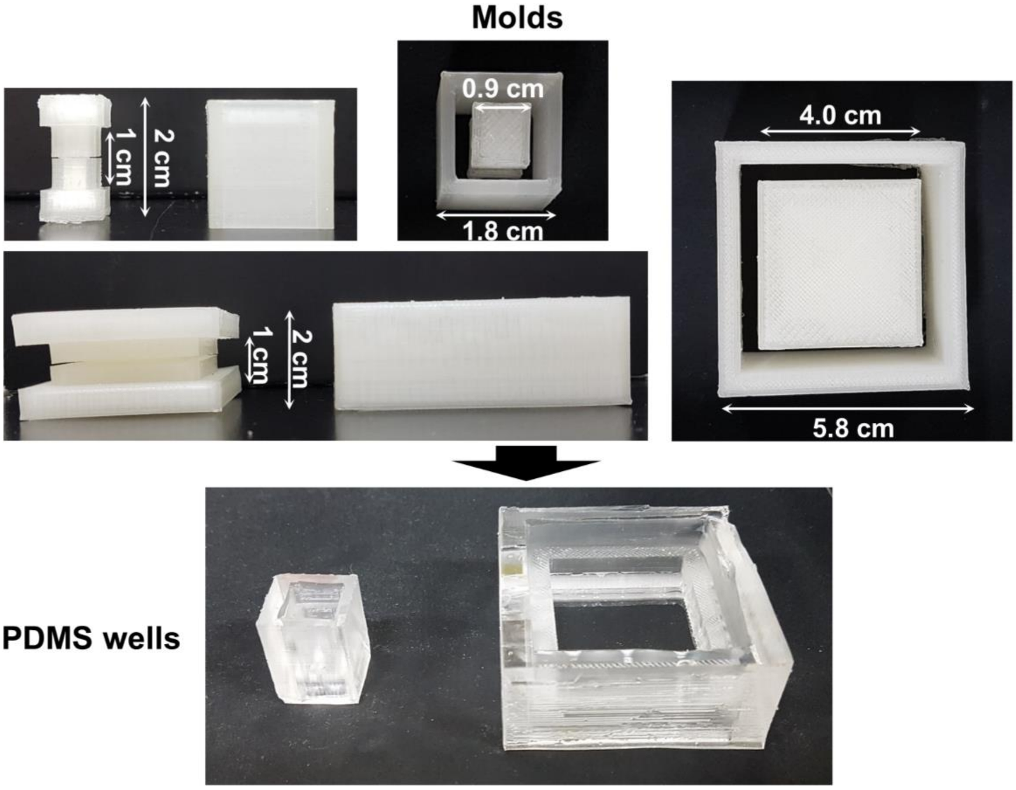

Supplementary Fig. 13 The fabrication of PDMS wells for TIP. The optical images of PDMS wells (lower) through the molds fabricated by FDM 3D printer (upper).

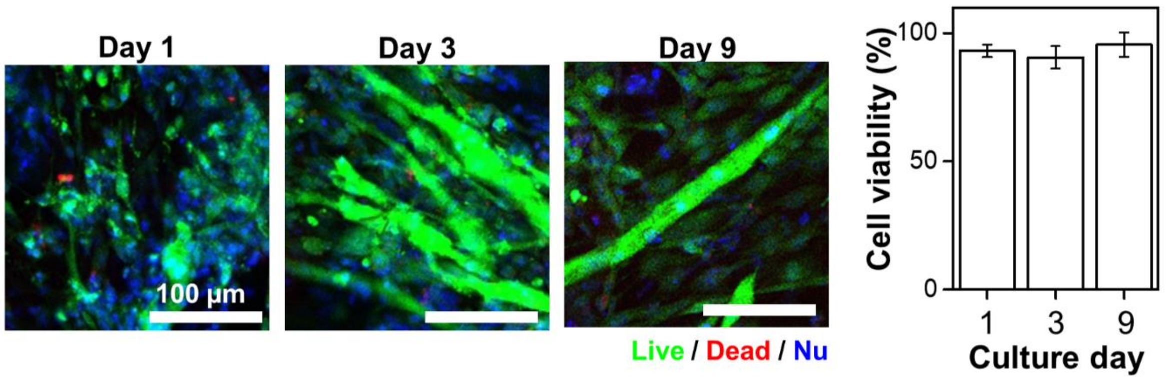

Supplementary Fig. 14 Cell viability of bSC printed by TIP. Error bars represents mean ± s.d. (n = 6 or 7 independent areas examined over independent 2 or 3 samples, green: live cells, red: dead cells, and blue: nucleus). Source data are provided as a Source Data file.

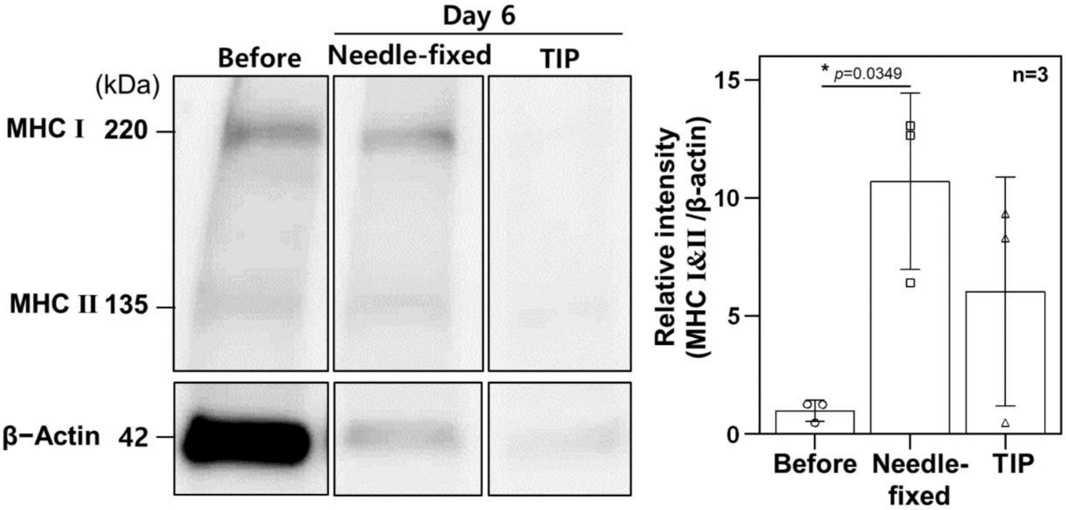

Supplementary Fig. 15 Western blot analysis of bSCs. Proteins were detected by using MHC (I&II) and β-actin antibodies (n = 3 independent samples, unpaired one-way ANOVA with a Tukey's HSD post-test). **p*<0.05 compared to before printing. Error bars represents mean ± s.d. Source data are provided as a Source Data file.

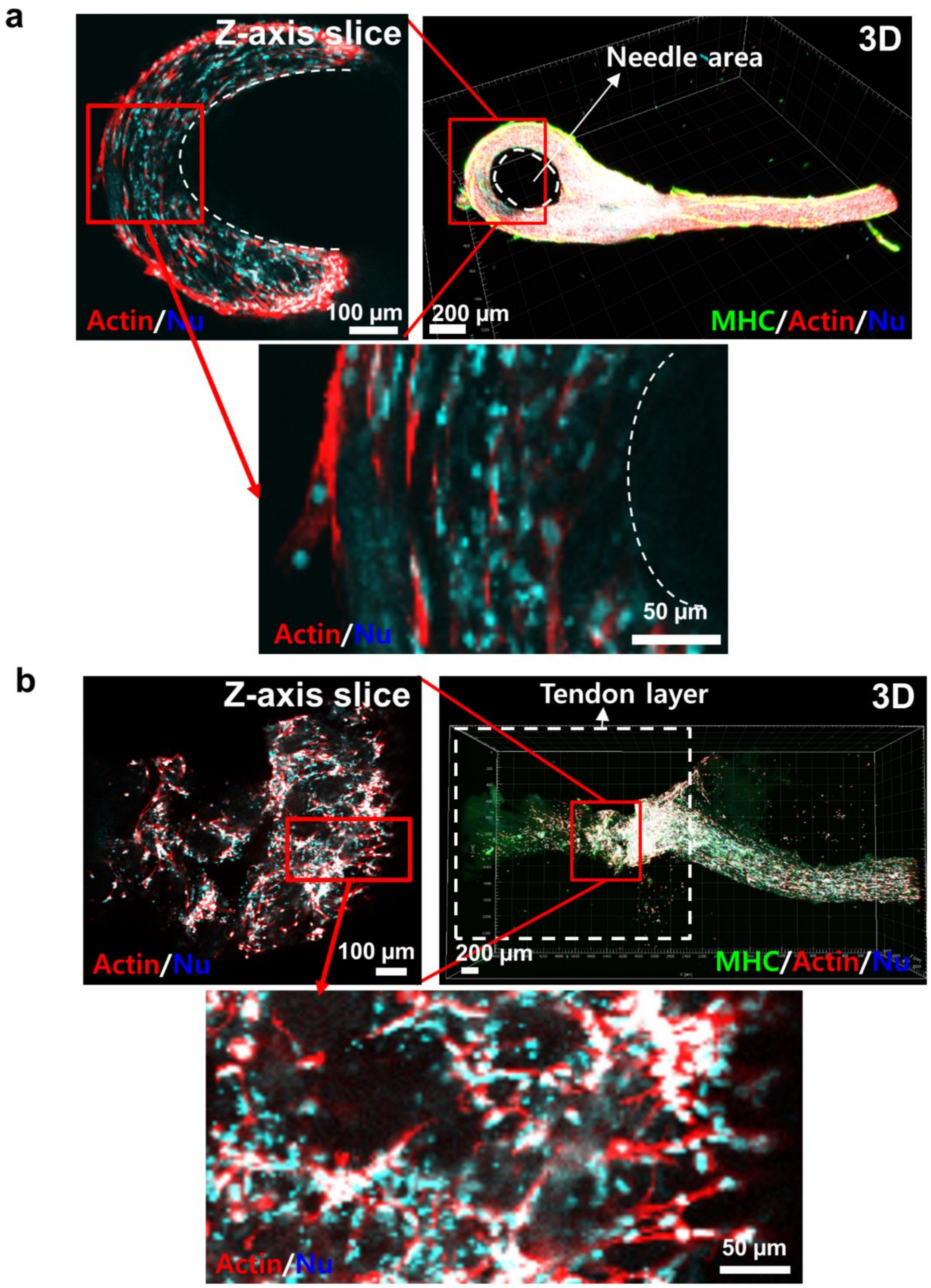

Supplementary Fig. 16 Comparison of connection areas in needle fixed culture and TIP. Fluorescence images (green: MHC, red: actin, and blue: nucleus) of bSCs tissues in needle fixed **(a)** culture and TIP **(b)**. Representative images from two independent experiments are shown.

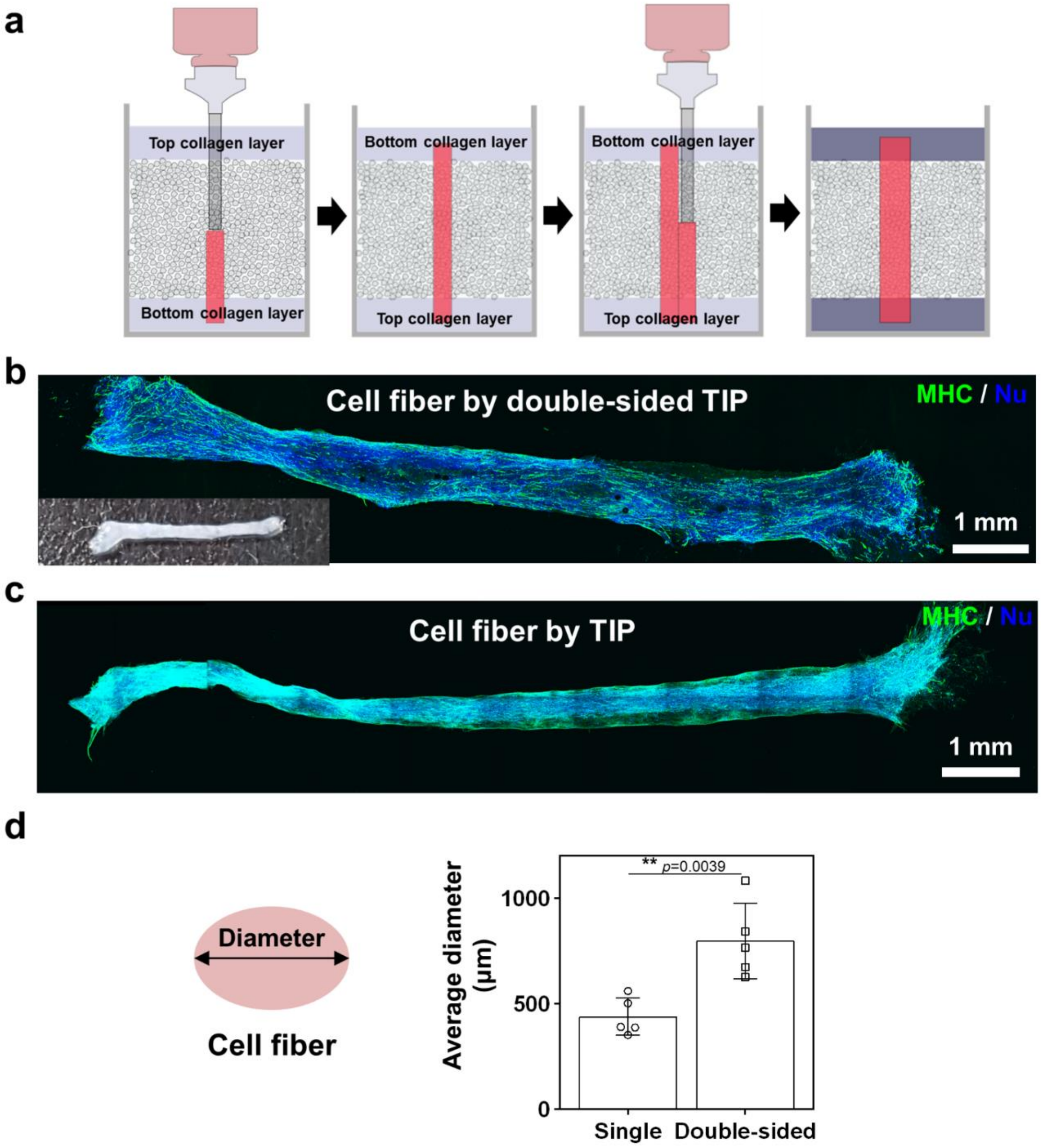

Supplementary Fig. 17 bSCs fiber fabricated by double-sided TIP. a, Schematic illustration of double-sided TIP, in which first printing was conducted same as general TIP and second printing was conducted at close to first printing area after flipping of the PDMS well. The two cell fibers fused into one fiber during culture. b-c, Z-projected fluorescence (green: MHC & blue: nucleus) and optical (inset) images of bSCs fiber formed by double-sided TIP on 7 day of differentiation (b) and bSCs fiber by one TIP printing on day 4 of differentiation. d, The diameters of cell fibers at 5 different spots (n=5 independent regions over 1 cell fiber, a pairwise t-test comparison). *$p<0.05$ compared to before printing. Error bars represents mean ± s.d. Representative images from at least three independent experiments are shown. Source data are provided as a Source Data file.

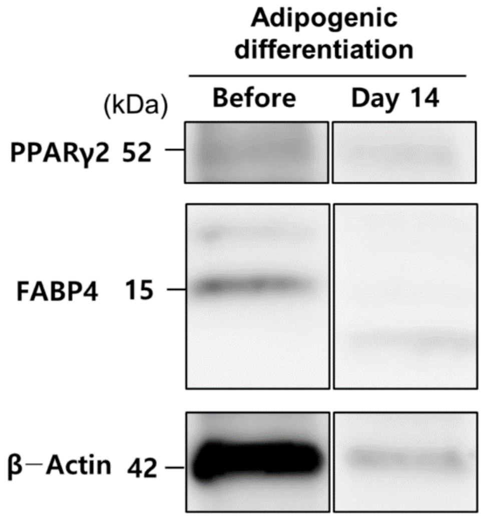

Supplementary Fig. 18 Western blot analysis of bADSCs. Proteins were detected by using PPARγ2, FABP4, and β-actin antibodies. Source data are provided as a Source Data file.

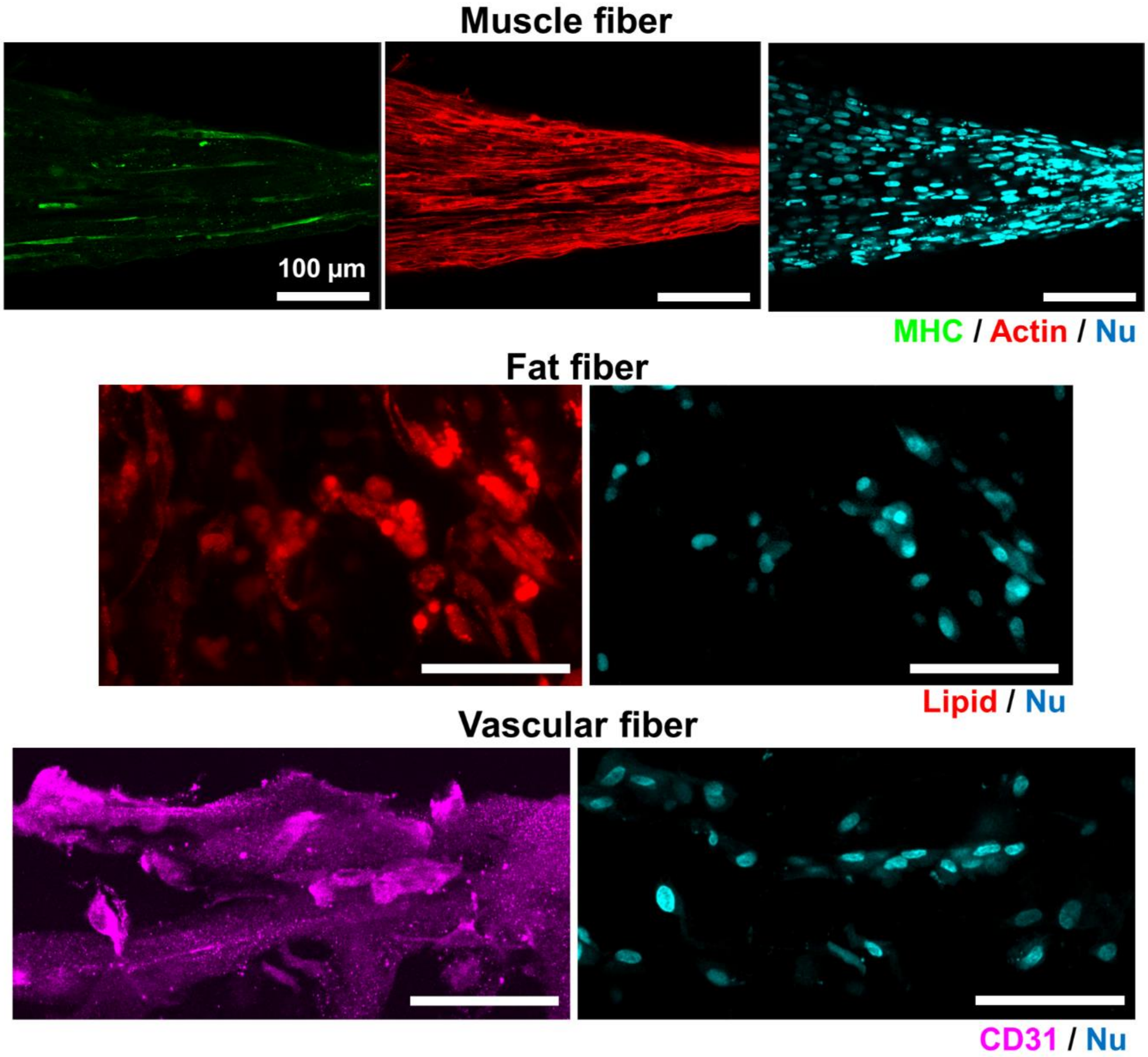

Supplementary Fig. 19 Single-channel fluoresce images of muscle, fat, vascular fibers fabricated by TIP. Muscle fiber on day 4 of differentiation (top, green: MHC, red: actin, and blue: nucleus), fat fiber on day 14 of differentiation (middle, red: lipid, and blue: nucleus), and vascular fiber on day 7 (bottom, magenta: CD31 and blue: nucleus). Representative images from at least three independent experiments are shown.

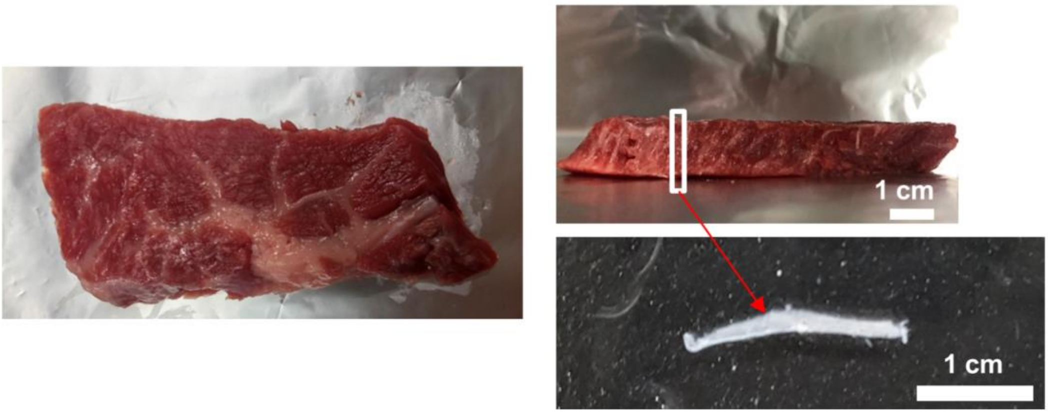

Supplementary Fig. 20 Optical images of commercial meat. The color of the collected fibrous tissue from commercial meat was decolorized during the 4% paraformaldehyde fixation.

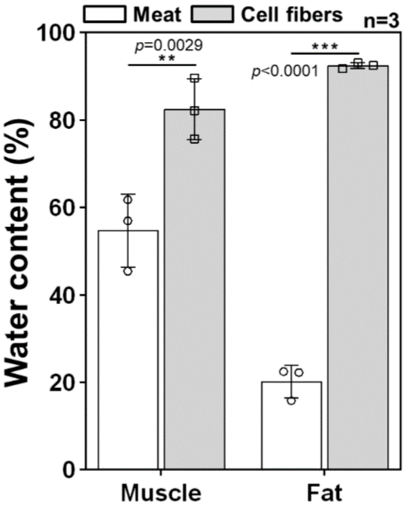

Supplementary Fig. 21 Water content measurement of muscle and fat cell fiber from commercial meat and TIP-derived. The weight measurement was conducted before and after freeze drying of fixed cell fibers (n=3 independent samples, pairwise t-test comparison). *p<0.05 and ***p<0.001; error bars represent mean ± s.d. Source data are provided as a Source Data file.

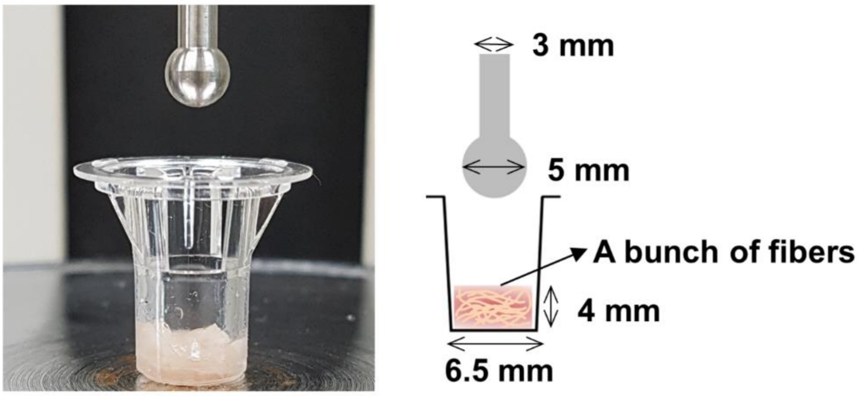

Supplementary Fig. 22 Compressive modulus measurement set-up. The measurement was conducted after pressing the cell fibers and fibrous tissues in the cup.

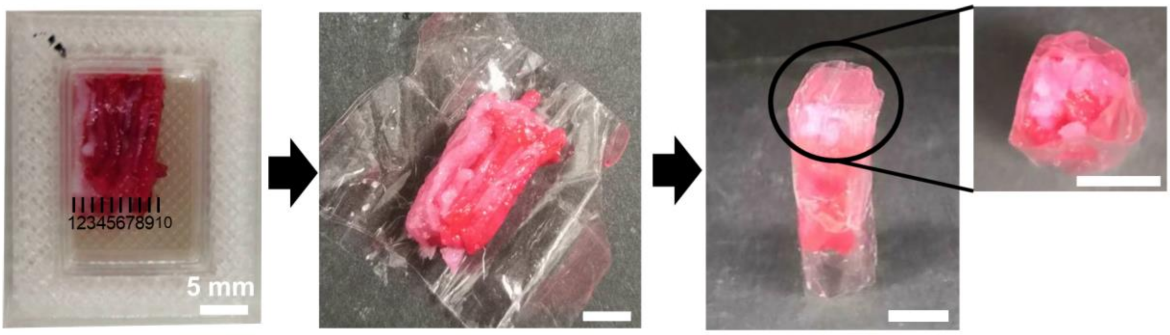

Supplementary Fig. 23 Bovine cell fiber assembly. After putting the cell fibers on a plastic container, treated by transglutaminase solution (10 U/mL), wrapped up with a plastic wrap, and kept at 4 °C for 2 days.

Supplementary Table. 1 List of antibodies used in this work

Antibody	Clone	Source	Dilution	Analysis
APC mouse anti-CD29	TS2/16	BioLegend, 303008	1:40	FACS sorting
PE-CyTM7 mouse anti-CD56	NCAM16.2	BD, 335826	1:40	FACS sorting
FITC mouse anti-CD31	CO.3E1D4	BIO-RAD, MCA1097F	1:40	FACS sorting
FITC mouse anti-CD45	1.11.32	BIO-RAD, MCA2220F	1:40	FACS sorting
Mouse anti-sarcomeric alpha actinin	EA-53	Abcam, ab9465	1:1000	Immunohistochemistry
Rabbit anti-Laminin	polyclonal	Sigma-Aldrich, L9393	1:100	Immunohistochemistry
Mouse anti-CD31	JC70A	Wako, M0823	1:100	Immunofluorescence staining
Rabbit anti-PPAR gamma	polyclonal	Abcam, ab45036	1:100 for IF and 1:1000 for WB	Immunofluorescence staining and Western Blot
Rabbit anti-FABP4	polyclonal	LSBio, LS-B4227	1:100 for IF and 1:1000 for WB	Immunofluorescence staining and Western Blot
Mouse anti-myosin 4	MF20	eBioscience, 14-6503-82	1:500 for IF and 1:1000 for WB	Immunofluorescence staining and Western Blot
Mouse anti-β-Actin	AC-15	Sigma-Aldrich, A5441	1:3000	Western Blot
Anti-Mouse IgG (H+L) Cross-Adsorbed Secondary Antibody Alexa Fluor 488	polyclonal	Invitrogen, A-11001	1:200	Immunofluorescence staining
Anti-Mouse IgG (H+L) Cross-Adsorbed Secondary Antibody Alexa Fluor 647	polyclonal	Invitrogen, A-21235	1:200	Immunofluorescence staining
Anti-Mouse IgG conjugated to horseradish peroxidase	polyclonal	BIORAD, 170-6516	1:5000	Western Blot
Anti-Rabbit IgG conjugated to horseradish peroxidase	polyclonal	BIORAD, 170-6515	1:5000	Western Blot

Supplementary Table. 2 List of Taqman probes used for real time qPCR analyses

Probe	Gene name	Assay ID (in Thermo Fisher Scientific)
Bovine myosin heavy chain II	*MYH2*	Bt03223147_gH
Bovine fatty acid binding protein 4	*FABP4*	Bt03213820_m1
Bovine platelet and endothelial cell adhesion molecule 1	*PECAM1*	Bt03215106_m1
Bovine peroxisome proliferator activated receptor gamma	*PPARG*	Bt03217547_m1
Bovine peptidylprolyl isomerase A	*PPIA*	Bt03224615_g1
