# Supplementary material for: Engineered whole cut meat-like tissue by the assembly of cell fibers using tendon-gel integrated bioprinting
Source: Nat Commun. 2021 Aug 24;12:5059. doi: 10.1038/s41467-021-25236-9 (PMC8385070; doi:10.1038/s41467-021-25236-9)
Supplement: Supplementary file 2 — Description of Additional Supplementary Files [file 41467_2021_25236_MOESM2_ESM.pdf]

Description of Additional Supplementary Files

Title: Supplementary Movie 1

Description: Supporting bath-assisted 3D printing of bovine satellite cells inside granular gellan gum bath

Title: Supplementary Movie 2

Description: Supporting bath-assisted 3D printing of bovine satellite cells inside granular gelatin bath

Title: Supplementary Movie 3

Description: Tendon-gel integrated bioprinting of bovine satellite cells

Title: Supplementary Movie 4

Description: 3D image of the tissue of the bovine satellite cells by tendon-gel integrated bioprinting on 3 day of differentiation (green: MHC, red: actin, and blue: nucleus)

Title: Supplementary Movie 5

Description: Multiple tissue printing by tendon-gel integrated bioprinting

Title: Supplementary Movie 6

Description: Whole 3D image of the muscle fiber by tendon-gel integrated bioprinting on 4 day of differentiation. (green: MHC & blue: nucleus)

Title: Supplementary Movie 7

Description: Whole 3D image of the fat tissue by tendon-gel integrated bioprinting on day 7. (red: lipid & blue: nucleus)

Title: Supplementary Movie 8

Description: Whole 3D image of the vascular tissue by tendon-gel integrated bioprinting on day 7 of differentiation. (red: CD31 & blue: nucleus)
