# Supplementary material for: Engineered whole cut meat-like tissue by the assembly of cell fibers using tendon-gel integrated bioprinting
Source: Nat Commun. 2021 Aug 24;12:5059. doi: 10.1038/s41467-021-25236-9 (PMC8385070; doi:10.1038/s41467-021-25236-9)
Supplement: Supplementary file 11 — Reporting Summary [file 41467_2021_25236_MOESM11_ESM.pdf]

nature research

Corresponding author(s): Michiya Matsusaki

Last updated by author(s): Jul 20, 2021

Reporting Summary

Nature Research wishes to improve the reproducibility of the work that we publish. This form provides structure for consistency and transparency in reporting. For further information on Nature Research policies, see our Editorial Policies and the Editorial Policy Checklist.

Statistics

For all statistical analyses, confirm that the following items are present in the figure legend, table legend, main text, or Methods section.

n/a	Confirmed	
☐	☒	The exact sample size (*n*) for each experimental group/condition, given as a discrete number and unit of measurement
☐	☒	A statement on whether measurements were taken from distinct samples or whether the same sample was measured repeatedly
☐	☒	The statistical test(s) used AND whether they are one- or two-sided<br>*Only common tests should be described solely by name; describe more complex techniques in the Methods section.*
☐	☒	A description of all covariates tested
☐	☒	A description of any assumptions or corrections, such as tests of normality and adjustment for multiple comparisons
☐	☒	A full description of the statistical parameters including central tendency (e.g. means) or other basic estimates (e.g. regression coefficient) AND variation (e.g. standard deviation) or associated estimates of uncertainty (e.g. confidence intervals)
☐	☒	For null hypothesis testing, the test statistic (e.g. *F*, *t*, *r*) with confidence intervals, effect sizes, degrees of freedom and *P* value noted<br>*Give P values as exact values whenever suitable.*
☒	☐	For Bayesian analysis, information on the choice of priors and Markov chain Monte Carlo settings
☒	☐	For hierarchical and complex designs, identification of the appropriate level for tests and full reporting of outcomes
☒	☐	Estimates of effect sizes (e.g. Cohen's *d*, Pearson's *r*), indicating how they were calculated

Our web collection on statistics for biologists contains articles on many of the points above.

Software and code

Policy information about availability of computer code

Data collection	ImageJ software (Fiji, version 1.52s, National Institute of Health, Bethesda, MD, USA) and Imaris software (version 9.3.1, Bitplane, Zurich, Switzerland) were used.
Data analysis	For the analysis, Excel from the Microsoft Office Professional Plus 2019, version 1808 was used. For the statistical analysis, Origin (version 9.0, OriginLab, Massachusetts, USA) and EzAnova software (version 0.98, University of South Carolina, Columbia, SC, USA) were used, as well as GraphPad Prism 8 (San Diego, USA).

For manuscripts utilizing custom algorithms or software that are central to the research but not yet described in published literature, software must be made available to editors and reviewers. We strongly encourage code deposition in a community repository (e.g. GitHub). See the Nature Research guidelines for submitting code & software for further information.

Data

Policy information about availability of data

All manuscripts must include a data availability statement. This statement should provide the following information, where applicable:

- Accession codes, unique identifiers, or web links for publicly available datasets
- A list of figures that have associated raw data
- A description of any restrictions on data availability

All source data are provided with this paper. All other data supporting the key findings of this study are available from the corresponding author upon reasonable request.

Field-specific reporting

Please select the one below that is the best fit for your research. If you are not sure, read the appropriate sections before making your selection.

- [x] Life sciences
- [ ] Behavioural & social sciences
- [ ] Ecological, evolutionary & environmental sciences

For a reference copy of the document with all sections, see nature.com/documents/nr-reporting-summary-flat.pdf

Life sciences study design

All studies must disclose on these points even when the disclosure is negative.

Sample size	No power analysis of sample size was done before performing the experiments. As the study relies on new methods, there were no data to compare with in order to determine the suitable sample size. To ensure reliable data we decided to perform 3-5 independent experiments with 1-12 replicates samples per condition per experiment and 1-4 pictures per sample for the image analysis. For the statistical analysis then, the average of the data measured in a same sample was taken and the mean of these average corresponding to one independent experiment was compared then to the other independent experiments averages. Given the reproducibility within and across the experiments, we got enough significant differences which enabled us to conclude that the sample size in our experiments were enough.
Data exclusions	The data from a broken sample by a handling fault were excluded from the analysis.
Replication	In each independent experiment (meaning different tissue donors), replicates were performed, with 1-12 replicates samples per condition per experiment and 1-4 pictures per sample for the image analysis. All attempts of replication succeeded and were included in the data. Only the data of the broken sample was excluded.
Randomization	Randomization methods did not apply to this study as there were no clinical populations or patients involved. For the fluorescence quantification, when only part of the samples were analyzed, the location of the images involved in the analysis was chosen randomly inside the sample area.
Blinding	Blinding did not apply to this study as there were no clinical population or patients involved. The investigators were not blinded during the data acquisition. But since the data sets are based on objectively measured data (fluorescent intensity, fluorescence area, western blot bands intensity, PCR...), blinding does not affect these data values.

Reporting for specific materials, systems and methods

We require information from authors about some types of materials, experimental systems and methods used in many studies. Here, indicate whether each material, system or method listed is relevant to your study. If you are not sure if a list item applies to your research, read the appropriate section before selecting a response.

Materials & experimental systems

n/a	Involved in the study	
	x	Antibodies
x		Eukaryotic cell lines
x		Palaeontology and archaeology
x		Animals and other organisms
x		Human research participants
x		Clinical data
x		Dual use research of concern

Methods

n/a	Involved in the study	
x		ChIP-seq
	x	Flow cytometry
x		MRI-based neuroimaging

Antibodies

Antibodies used

Conjugated first antibodies for FACS:
APC mouse anti-CD29 (BioLegend, 303008, TS2/16, dilution 1/40)
PE-CyTM7 mouse anti-CD56 (BD, 335826, NCAM16.2, dilution 1/40)
FITC mouse anti-CD31 (BIO-RAD, MCA1097F, CO.3E1D4, dilution 1/40)
FITC mouse anti-CD45 (BIO-RAD, MCA2220F, 1.11.32, dilution 1/40)

First antibodies:
Mouse anti-sarcomeric alpha actinin (Abcam, ab9465, EA-53, dilution 1/1000)
Rabbit anti-Laminin (Sigma-Aldrich, L9393, polyclonal, dilution 1/1000)
Mouse anti-CD31 (Wako, M0823, JC70A, dilution 1/100)
Rabbit anti-PPAR gamma (Abcam, ab45036, polyclonal, Lot GR3304468-3, dilution 1/100 for IF, 1/1000 for WB)
Rabbit anti-FABP4 (LSBio, LS-B4227, polyclonal, Lot 110145, dilution 1/100 for IF, 1/1000 for WB)
Mouse anti-myosin 4 (eBioscience, 14-6503-82, MF20, Lot 2265353, dilution 1/500 for IF, 1/1000 for WB)

Mouse anti-β-Actin (Sigma-Aldrich, A5441, AC-15, Lot 029M883V, dilution 1/3000)

Second antibodies:
Anti-Mouse IgG (H+L) Cross-Adsorbed Secondary Antibody Alexa Fluor 488 (Invitrogen, A-11001, polyclonal, dilution 1/200)
Anti-Mouse IgG (H+L) Cross-Adsorbed Secondary Antibody Alexa Fluor 647 (Invitrogen, A-21235, polyclonal, dilution 1/200)
Anti-Mouse IgG conjugated to horseradish peroxidase (BIORAD, 170-6516, Lot 64273614, dilution 1/5000)
Anti-Rabbit IgG conjugated to horseradish peroxidase (BIORAD, 170-6515, Lot 64371828, dilution 1/5000)
Precision ProteinTM StrepTactin-HRP Conjugate (BIORAD, 161-0380, Lot 64333457, dilution 1/5000)

Validation

APC mouse anti-CD29 (https://www.biolegend.com/ja-jp/global-elements/pdf-popup/apc-anti-human-cd29-antibody-852?filename=APC anti-human CD29 Antibody.pdf&pdfgen=true)
PE-CyTM7 mouse anti-CD56 (https://www.bdbiosciences.com/content/bdb/paths/generate-tds-document.us.335791.pdf)
FITC mouse anti-CD31 (https://www.bio-rad-antibodies.com/monoclonal/sheep-ovine-cd31-antibody-co-3e1d4-mca1097.html?f=purified#external-images)
FITC mouse anti-CD45 (https://www.bio-rad-antibodies.com/monoclonal/sheep-ovine-cd45-antibody-1-11-32-mca2220.html?f=purified)
Mouse anti-sarcomeric alpha actinin (https://www.abcam.com/sarcomeric-alpha-actinin-antibody-ea-53-ab9465.html)
Rabbit anti-Laminin (https://www.sigmaaldrich.com/JP/en/product/sigma/l9393)
Mouse anti-myosin 4 (https://www.thermofisher.com/antibody/product/Myosin-4-Antibody-clone-MF20-Monoclonal/14-6503-82)
Mouse anti-CD31 (https://www.agilent.com/en/product/immunohistochemistry/antibodies-controls/primary-antibodies/cd31-endothelial-cell-(concentrate)-76539)
Rabbit anti-PPAR gamma (https://www.abcam.com/ppar-gamma-antibody-ab45036.html)
Rabbit anti-FABP4 (https://www.lsbio.com/antibodies/ihc-plus-fabp4-antibody-ap2-antibody-tyr20-ihc-wb-western-ls-b4227/118621)
Mouse anti-β-Actin (https://www.sigmaaldrich.com/fr/product/sigma/a5441&usg=AOvVaw0y8OlGgNZoDLFtSJ7nqOG5)

Flow Cytometry

Plots

Confirm that:

- [x] The axis labels state the marker and fluorochrome used (e.g. CD4-FITC).
- [x] The axis scales are clearly visible. Include numbers along axes only for bottom left plot of group (a 'group' is an analysis of identical markers).
- [x] All plots are contour plots with outliers or pseudocolor plots.
- [x] A numerical value for number of cells or percentage (with statistics) is provided.

Methodology

Sample preparation

Bovine satellite cells (bSCs) were isolated from 160 g fresh masseter muscle samples (within 6 h of euthanasia) of 27-month-old Japanese black cattle obtained at the slaughterhouse (Tokyo Shibaura Zouki, Tokyo, Japan, and JA ZEN-NOH Kanagawa, Kanagawa, Japan). The freshly harvested bovine muscle was kept on ice, transferred to a clean bench, and washed with cold 70 % ethanol for 1 min, followed by cold PBS1x 2 times. Then, the fat tissue part was disposed of, the remaining muscle was cut into small pieces with a knife, and minced with a food processor mechanically. The bovine minced muscle was washed with cold PBS1x with 1 % penicillin-streptomycin (PS) (Lonza, 17-745E) for 1 min. The washed muscle was transferred to a bottle and mixed with 160 ml of 0.2 % collagenase II (Worthington, CLS-2) in DMEM (Invitrogen, 41966-29) supplemented with 1 % PS. The bottle was incubated and shaken every 10 min for 1.5 h at 37 °C. After digestion, 160 mL 20 % FBS in DMEM supplemented with 1 % PS was added and mixed well. The mixed solution was centrifuged for 3 min at 80 g and 4 °C. Floating tissues in the supernatant after centrifugation was removed by tweezers and then collected supernatant kept on ice as a mononuclear cell suspension. Precipitated debris was mixed with 80 ml cold 1 % PS in PBS1x and centrifuged for 3 min at 80 g and 4 °C. The supernatant was collected again and mixed with the former mononuclear cell suspension. After that, the cells were filtered through a 100 µm cell strainer. After centrifugation for 5 min at 1,500 g and 4 °C, the cells were suspended with 160 ml cold DMEM with 20 % FBS and 1 % PS, were filtered through a 100 µm cell strainer followed by a 40 µm cell strainer. The cells were then centrifuged for 5 min at 1,500 g and 4 °C. Precipitated cells were incubated with 8 ml erythrocyte lysis buffer (ACK, 786-650) for 5 min on ice. Then the cells were washed twice with cold PBS1x supplemented with 1 % PS and the cell pellet was mixed with FBS supplement with 10 % dimethyl sulfoxide and then reserved at -150 °C. The frozen cells were recovered in a 37 °C water bath and washed with cold PBS1x twice. The cells were suspended in F10 medium (Gibco, 31550-023) containing 20 % FBS, 5 ng/mL bFGF (R&D, 233-FB-025) and 1 % PS supplemented with 10µM p38i (Selleck, S1076), and then seeded at 1.1x105 cells/well in 6-well cell culture plates (Corning) that were coated with 0.05 % bovine collagen type I (Sigma, C4243). The cells were cultured by changing the medium every two or three days and were passaged when they reach 60 % of confluency until passage 3 for cell sorting by flow cytometry.

Instrument

SH800S Cell Sorter (Sony Biotechnology)

Software

The FlowJo software was used in FACS data analysis.

Cell population abundance

The population abundance of bovine satellite cells (CD31-CD45-CD29+CD56+ cells) were provided in Supplementary Fig. 1.

Gating strategy	The gating strategy for bovine satellite cells (CD31-CD45-CD29+CD56+ cells) were provided in Supplementary Fig. 1.

☒ Tick this box to confirm that a figure exemplifying the gating strategy is provided in the Supplementary Information.
